# An energy-aware diffusion graph neural network based on intuitionistic fuzzy credibility graphs for research-paper classification

**DOI:** 10.3389/frai.2026.1973276

**Published:** 2026-10-23

**Authors:** Sadhu Varun, Shaik Sharief Basha

**Affiliations:** 1 Department of Mathematics, School of Advanced Sciences, Vellore Institute of Technology, Vellore, Tamil Nadu, India; 2 Department of Analytics, School of Computer Science and Engineering, Vellore Institute of Technology, Vellore, Tamil Nadu, India

**Keywords:** credibility of membership, credibility of non-membership, diffusion, graph neural networks, intuitionistic fuzzy credibility graph, intuitionistic fuzzy credibility relation, node-based Laplacian energy

## Abstract

The classification of research papers from different academic data is still challenging due to the presence of semantic ambiguity, lack of information, fluctuating credibility, and complex linkages across research articles. Existing fuzzy graph neural networks depend too much on membership information and cannot truly consider the intuitionistic uncertainty, trust dependability, and structural importance of nodes at the graph propagation level. To overcome these constraints, this paper presents an intuitionistic fuzzy graph neural network architecture that combines trust-aware intuitionistic representations and a unique node Laplacian energy mechanism. The suggested Node Laplacian Energy is derived from the graph Laplacian's row-wise norm and is used as an adaptive node-weighting metric to assess each research paper's local structural contribution. Furthermore, a diffusion-based propagation technique is used to enhance information transmission across semantically linked publications and capture larger graph relationships. The suggested system is tested for research-paper categorization with semantic similarity features and intuitionistic fuzzy credibility information. Its performance is compared to known machine learning and graph neural network baselines utilizing accuracy, precision, recall, and F1-score. The findings show that including uncertainty-aware representations, structural node weighting, and diffusion-based graph propagation can improve discriminative academic content categorization. The suggested paradigm offers a unified way to model semantic linkages, uncertainty, believability, and graph structural relevance in research-paper categorization.

## 1 Introduction

Fuzzy logic and fuzzy sets were first introduced by Zadeh (1965). Ambiguity and uncertainty are studied in different contexts such as artificial intelligence (AI), business, and healthcare. The concept of fuzzy sets was introduced by Atanassov (2016), and then the notion of intuitionistic fuzzy sets (IFS) was developed based on the concept of fuzzy sets by adding a new degree called non-membership to address the ambiguity and uncertainty of the membership degree. This generalization of classical fuzzy sets considers both the membership and non-membership degrees simultaneously and provides a better framework for handling uncertainty compared to classical fuzzy theory (Atanassov, 2016). The concept of Intuitionistic Fuzzy Graph (IFG) was introduced by researchers using the concept of intuitionistic fuzzy sets, where uncertainty has been incorporated into the structure of graphs by associating intuitionistic fuzzy data to vertices and edges, thus making IFGs suitable for modeling complex real-world systems (Akram et al., 2014; Parvathi et al., 2009). The fuzzy credibility set is proposed in 2020 to demonstrate the trustworthiness of fuzzy evaluation data (Ye et al., 2021a). In uncertain conditions, it is important to increase the credibility of fuzzy intuitionistic ideas and adjust the values of suggestions. To overcome the limits of processing ambiguous judgments, researchers recommended adding credibility degrees to the intuitionistic fuzzy idea. To improve the reliability of fuzzy assessment, the intuitionistic fuzzy credibility set was proposed by researchers, which combines credibility with membership and non-membership information (Ye et al., 2021b).

Graph energy is a classical spectral graph invariant that specifies structural features of a graph by means of eigenvalues of its adjacency matrix. Gutman (1978) defined the graph energy as the sum of the absolute values of the eigenvalues of the adjacency matrix, a remarkable relationship between the structure of a graph and its spectral features. The use of Laplacian energy necessitates distinguishing between graph-level spectral metrics and node-level structural descriptors. The classical Laplacian energy measures the global structure of a graph by the spectrum of the Laplacian matrix (Gutman and Zhou, 2006; Merris, 1994; Brouwer and Haemers, 2012). The concept of Laplacian energy was defined for intuitionistic fuzzy graphs (Praba et al., 2018; Noorjahan and Basha, 2025; Basha and Kartheek, 2015) before. More recently, Guerrero (2025; p. 43) proposed Laplacian and normalized-Laplacian energies connected to individual vertices, establishing links between vertex energy and combinatorial, spectral, and geometric features. These findings provide a mathematical foundation to study the structural energy at the node level. In the current study, however, the purpose is not to propose a new matrix norm or redefine traditional Laplacian energy. Instead, the node-level quantity obtained from the Laplacian matrix is adopted as an explicit structural description in an intuitionistic credibility graph and then plugged into the neural representation learning. This difference distinguishes the proposed use of NBLE from previous mathematical investigations of Laplacian energy.

Graph neural networks (GNNs) are presented as a powerful deep learning method for learning on graph-structured material by pooling information from surrounding nodes (Goodfellow et al., 2016). The Graph Convolutional Network (GCN) proposed by Kipf and Welling (2016) has shown to be an excellent spectral-based graph convolution framework for semi-supervised node categorization. GraphSAGE (Hamilton et al., 2017) generalizes this idea by inductive neighborhood aggregation, while Graph Attention Networks (GAT) (Veličković et al., 2018) uses attention processes to provide variable significance to surrounding nodes. Later studies have provided thorough reviews of the designs, applications, and theory of GNNs (Zhou et al., 2020; Bronstein et al., 2021). Benchmarking studies have also emphasized the need for uniform experimental evaluation across GNN designs (Lemos, 2023; Feng et al., 2020). Recent studies have generalized GNNs for text and document categorization showing their capacity to leverage links between documents and characteristics (Wang et al., 2024).

Recent efforts have also started to include fuzzy representations directly into graph neural networks. Zhang et al. (2023) suggested fuzzy representation learning on graphs. Lin et al. (2024) offered FuzzyGCN, which is representation learning on uncertain graphs by embedding fuzzy numbers into neural weights and biases. These works illustrate that fuzzy uncertainty may be included in graph representation learning and GNN parameters. However, these techniques do not explicitly build an intuitionistic credibility graph where membership, non-membership, and credibility information are simultaneously determining the graph representation. The proposed IFCG-GNN differentiates at this level by adding credibility-aware intuitionistic information into graph building prior to Laplacian-based structural encoding and diffusion-based neural propagation.

Diffusion-based graph learning offers an alternate technique to propagate information outside the traditional local neighborhood aggregation. Diffusion-Convolutional Neural Networks (DCNNs) (Atwood and Towsley, 2016) proposed diffusion-based representations for graph node categorization, and Diffusion Convolutional Recurrent Neural Networks (DCRNNs) (Li et al., 2017) expanded diffusion convolution to spatio-temporal prediction. More recent works have examined diffusion as a principled neural propagation process. GREAD proposes a novel graph representation learning approach based on the reaction-diffusion equations and especially tackles the over-smoothing problem with diffusion-based propagation (Choi et al., 2023). Li et al. (2024) established an extended neural diffusion framework and incorporated high-order diffusion to use information from higher-order neighbors. More recently, diffusion-Jump GNNs have proposed learnable diffusion distances and structural filters to form non-local interactions between nodes, especially for heterophilic graphs (Begga et al., 2025; Sun et al., 2025). These studies highlight the potential of diffusion-based propagation to enhance information transmission across graph nodes. However, existing diffusion methods mostly work on crisp node representations and do not explicitly consider intuitionistic fuzzy information or node-level Laplacian energy.

Sentence-BERT permits to compute embeddings of scientific articles that are semantically meaningful and the comparison of research papers based on the text content may be performed efficiently (Reimers and Gurevych, 2019). OpenAlex offers a wide scale academic metadata and citation network to enable graph-based research-paper categorization and assessment of graph learning algorithms (Priem et al., 2022). The matrix row 1-norm is an important measure in numerical equations that describes the size and structural properties of a matrix. It is a short measure based on the absolute values of the matrix members. It is useful for determining the size, stability, and numerical behavior of matrix (Golub and Van Loan, 2013). The 1-norm is computationally inexpensive and can be useful for large matrices (Van Loan, 1996). Neymeyr et al. (2021) studied the signal contribution function as a function of various matrix norms. They reconsidered the summed-signal approach originally introduced by Gemperline and extended by Tauler. Neymeyr et al. showed that the entry wise 1-norm provides mathematically meaningful bounds for two-component systems and possibly better interpretability through summed signal contributions.

### 1.1 Limitations

Current methodologies for classifying research papers have progressively integrated graph-based representations to leverage the interconnections of research articles, citations, and semantic affinities. Traditional machine learning and deep learning approaches often model documents in isolation or rely on rigid graph structures that model relations with binary or scalar-valued edges. Although GNN architectures such as GCN, GraphSAGE, and GAT are effective in aggregating information from neighboring nodes, they generally do not transfer the uncertainty and reliability directly associated with the links between research publications (Wang et al., 2024; Haider Rizvi et al., 2025; Corso et al., 2024). Intuitionistic fuzzy graphs handle ambiguity through membership and non-membership information, but their integration with neural graph classification and explicit credibility is scarce (Razzaque et al., 2023; Talebi and Rashmanlou, 2024). Further, credibility-aware fuzzy models describe the reliability of uncertain evaluations and have been mostly used for similarity, aggregation, and decision-making issues (Ye et al., 2021a,b; Ullah et al., 2025). Recent work on Laplacian energy provides structural information at graph or vertex levels (Praba et al., 2018; Basha and Kartheek, 2015; Guerrero, 2025). However, its use in credibility-aware neural graph learning is still restricted. Meanwhile, diffusion-based GNNs have studied reaction-diffusion, generalized diffusion, and diffusion-jump propagation (Begga et al., 2025; Choi et al., 2023; Li et al., 2024). These techniques show the benefit of long-range information transmission but do not explicitly include intuitionistic credibility with the node-level Laplacian structural information. Thus, there exists a methodological gap to jointly describe uncertainty, credibility, structural energy, and diffusion-based propagation in a unified graph-learning framework for research-paper categorization.

### 1.2 Research gap

The recent literature can be roughly categorized into four directions: conventional GNNs for graph-based representation learning (Kipf and Welling, 2016; Hamilton et al., 2017; Veličković et al., 2018; Zhou et al., 2020; Bronstein et al., 2021; Wang et al., 2024; Haider Rizvi et al., 2025; Corso et al., 2024), fuzzy GNNs for uncertainty-aware representation and message passing (Zhang et al., 2023; Lin et al., 2024), intuitionistic fuzzy graphs for membership, non-membership, and Laplacian-based structural analysis (Praba et al., 2018; Noorjahan and Basha, 2025; Basha and Kartheek, 2015; Razzaque et al., 2023; Talebi and Rashmanlou, 2024; Guerrero, 2025), and diffusion-based GNNs for extended information propagation (Begga et al., 2025; Choi et al., 2023; Li et al., 2024). These techniques, however, only solve their parts individually. Fuzzy GNNs are concerned with fuzzy representations or propagation, while intuitionistic fuzzy graph research is focused on mathematical characteristics and decision-making. Similarly, credibility-based fuzzy models essentially enable uncertainty-aware evaluation and decision-making (Ye et al., 2021a,b; Ullah et al., 2025). Recent diffusion GNNs focus on receptive-field expansion, over-smoothing, or non-local propagation (Begga et al., 2025; Li et al., 2024). However, the combination of membership, non-membership, and credibility within graph building, node-level Laplacian structural encoding, and diffusion-based propagation has not been extensively examined for research-paper categorization. The proposed IFCG-GNN solves this issue by building a unified uncertainty-aware graph-learning system. It is worthy to remark that the contribution is not claimed to fuzzy logic, Laplacian energy, or diffusion separately, but for their methodological integration inside the suggested architecture. To justify the proposed framework concisely in relation to recent developments, in Table 1 we compare representative approaches according to their treatment of uncertainty, intuitionistic membership and non-membership, credibility, node-level Laplacian energy, diffusion propagation, and graph neural learning. The comparison underscores the methodological factors individually addressed in earlier research and the special integration studied in the proposed IFCG-GNN. For the sake of clarity, the following notation is used in Table 1: μ represents membership, γ represents non-membership, *C*_μ_ represents the credibility associated with membership, and *C*_γ_ represents the credibility associated with non-membership.

**Table 1:** Critical comparison of representative approaches.

Approach	Fuzzy	μ	γ	*C*_μ_	*C*_γ_	NBLE	Diffusion	GNN
IFG studies (Praba et al., 2018; Basha and Kartheek, 2015; Razzaque et al., 2023; Talebi and Rashmanlou, 2024; Guerrero, 2025)	Yes	Yes	Yes	No	No	Yes	No	No
Fuzzy credibility graph (Ullah et al., 2025)	Yes	Yes	Yes	Yes	No	No	No	No
Fuzzy representation learning (Zhang et al., 2023)	Yes	No	No	No	No	No	No	Yes
FuzzyGCN (Lin et al., 2024)	Yes	No	No	No	No	No	No	Yes
DCNN (Atwood and Towsley, 2016)	No	No	No	No	No	No	Yes	Yes
GREAD (Choi et al., 2023)	No	No	No	No	No	No	Yes	Yes
Generalized neural diffusion (Li et al., 2024)	No	No	No	No	No	No	Yes	Yes
DJ-GNN (Begga et al., 2025)	No	No	No	No	No	No	Yes	Yes
Proposed IFCG-GNN	Yes	Yes	Yes	Yes	Yes	Yes	Yes	Yes

As shown in Table 1, the current methods mostly treat uncertainty representation, intuitionistic fuzzy modeling, structural energy, credibility, or diffusion propagation as individual methodological issues. Thus, the research gap that this approach addresses is not only the absence of one component. Instead, it is the lack of a unified graph-learning formulation that (i) leverages uncertainty and credibility to define the graph representation, (ii) has node-level Laplacian information available as an explicit structural signal, and (iii) performs diffusion propagation on the resulting intuitionistic credibility graph. The suggested IFCG-GNN is aimed to explore this methodological interplay.

### 1.3 Motivation

The identified deficiency motivates the development of a graph learning framework that can jointly incorporate uncertainty, reliability, and structural properties of research-paper links. The degrees of membership and non-membership provide us with insight into the robustness of the affirmative and negative evidence, respectively, and their corresponding trust values provide more details about the dependability of these estimates. Thus, a four-element framework (μ, γ, *C*_μ_, *C*_γ_) is employed to store both evidentiary and credibility data, eliminating the necessity for a separate hesitation element. Moreover, the Laplacian energy at the node level can provide extra structural information by capturing the diversity or importance of certain nodes in the graph. Integrating diffusion propagation allows information to propagate through the graph, moving beyond just local interactions. The combination of these synergistic characteristics motivates us to develop a credibility- and energy-aware graph learning framework. Specifically, uncertainty-aware links, structural energy, and disseminated graph data work together to facilitate research-paper classification.

### 1.4 Proposed model

This work introduces a diffusion-based graph neural network framework for credibility-aware and energy-efficient research-paper categorization. The research articles are first modeled as nodes within a graph, and the edges are formed based on the chosen semantic and citation-based relationship parameters. A four-part credibility framework (μ, γ, *C*_μ_, *C*_γ_) is presented for the credibility of each node and its associated links, where μ and γ are the degrees of membership and non-membership, respectively, and *C*_μ_ and *C*_γ_ are the corresponding trust measures. The generated credibility data is presented in the graph to ensure the strength and reliability of the connections. Therefore, the graph Laplacian is used to extract node-specific energy information, which is then incorporated into the GNN training process to improve structural understanding. Then, a diffusion propagation method is proposed to enhance the information propagation of graph neighbors. Finally, the produced credibility-, energy-, and diffusion-aware representations are given to the GNN classifier to obtain discriminative representations for research-paper categorization.

### 1.5 Contributions

The main contributions of this work are summarized as follows:

1. Intuitionistic credibility representation: an intuitionistic credibility representation is developed for research-paper associations by jointly expressing membership, non-membership, and corresponding credibility information. This provides a generalization of representing uncertainty beyond typical scalar similarity or fuzzy membership alone.
2. Credibility-aware graph construction: the proposed approach encodes the credibility-aware representation into an explicit graph structure, where the interactions between research articles are learned from their uncertainty and credibility-aware representations. Thus, the uncertainty propagates over the graph topology employed in future neural propagation and not merely at the neural-parameter level.
3. Node-level Laplacian structural encoding: the node-based Laplacian energy (NBLE) is produced from the built graph as an explicit node-level structural description. NBLE is integrated into the initial node representation and further employed as a node-specific weighting mechanism in the propagation of hidden representations. The study does not provide a new matrix norm but instead studies the use of node-level Laplacian structural information in credibility-aware neural graph learning.
4. Credibility-aware diffusion propagation: a diffusion mechanism is used on the created intuitionistic credibility graph for information propagation beyond the usual local neighborhood aggregation. The originality here is not diffusion itself, which has been extensively explored (Atwood and Towsley, 2016; Choi et al., 2023; Li et al., 2024), but its integration with the suggested uncertainty- and credibility-aware graph model and the node-level structural encoding.
5. Unified research-paper classification framework: the resulting IFCG-GNN blends intuitionistic credibility representation, graph structural information, NBLE, and diffusion propagation into a unified framework of research-paper classification. We compare the framework with classical GNNs and current diffusion-based techniques and perform ablation and parameter-sensitivity experiments to disentangle the impacts of different components.

### 1.6 Framework of this study

The structure of the study paper is as follows: some previous notions of intuitionistic fuzzy credibility graphs and maximum absolute row sum are discussed in Section 2. In Section 3, we constructed an intuitionistic fuzzy credibility graph and proposed the node-based Laplacian energy. The problem statement is described in Section 4. In Section 5, we propose a unique IFCG-GNN technique using the diffusion layer and node-based Laplacian energy as the model weights. In Section 6, we use the algorithm of our proposed approach to acquire the results, compare with existing techniques, and examine the complexity and scalability of the proposed method. Conclusions and future work are discussed in Section 7.

## 2 Preliminaries

Definition 1: Fuzzy credibility graph (Ye et al., 2021a).

The fuzzy credibility graph G = (V, E) on a universal set T is defined as follows


$$\begin{array}{l} \;\mu_{ij}\left(tu\right)\leq \min\{\mu_{i}\left(t\right),\mu_{j}\left(u\right)\}\\ C_{\mu_{ij}}\left(tu\right)\leq \max\{C_{\mu }\left(t\right),C_{\mu }\left(u\right)\} \end{array}$$

Where φ = {*t*, μ_φ_(*t*), *C*_μ_(*t*)/*tϵT*} is the fuzzy credibility set on *T*, which represents the node-related fuzzy credibility values, and *r*_φ_ = {*tu*, μ_*r*_φ__(*tu*), *C*_*r*_φ__(*tu*)/*tu ϵ TXT*} is the fuzzy credibility relation relative to ϕ, which represents the edge-related fuzzy credibility values.

Definition 2: Intuitionistic fuzzy credibility set (Ye et al., 2021b).

The intuitionistic fuzzy credibility set on a universal set *T* is expressed as follows:


$$\begin{array}{l} \varphi^{\prime }=\left\{t,\left(\mu_{\varphi^{\prime }}\left(t\right),C_{\mu }\left(t\right)\right),\left(\gamma_{\varphi^{\prime }}\left(t\right),C_{\gamma }\left(t\right)\right)/t\epsilon T\right\}. \end{array}$$

Where $\left(\mu_{\varphi }\left(t\right),C_{\mu }\left(t\right)\right)\;:T\;\to {\left[0,1\right]}^{2}$ be the representation of membership and credibility of membership, and $\left(\gamma_{\varphi }\left(t\right),C_{\gamma }\left(t\right)\right)\;:T\;\to {\left[0,1\right]}^{2}$ be the notation of non-membership and credibility of non-membership, and φ′ represents the node-related intuitionistic fuzzy credibility values.

Definition 3: Maximum absolute row-sum norm (Golub and Van Loan, 2013).

The maximum absolute row-sum norm of a matrix *A* = [*a*_*ij*_], is defined as


$$\begin{array}{l} \|A{\|}_{\infty }=\;\underset{i}{\max}\underset{j}{\sum }|a_{ij}|. \end{array}$$

## 3 Intuitionistic fuzzy credibility graph

In this section we discuss the definition of suggested intuitionistic fuzzy credibility graph, adjacent matrix of IFCG, Laplacian matrix of IFCG and Node based Laplacian energy.

Definition 1: Intuitionistic fuzzy credibility relation.

Let $\left(\mu_{\varphi }\left(tu\right),C_{\mu }\left(tu\right)\right)\;:T\;\to {\left[0,1\right]}^{2}$ be the representation of membership and credibility of membership, and $\left(\gamma_{\varphi }\left(tu\right),C_{\gamma }\left(tu\right)\right)\;:T\;\to {\left[0,1\right]}^{2}$ be the notation of non-membership and credibility of non-membership; then the intuitionistic fuzzy credibility relation on IFCS φ′ in *T* × *T* is expressed as follows:


$$\begin{array}{l} r_{\varphi^{\prime }}=\left\{tu,\left(\mu_{r_{\varphi^{\prime }}}\left(tu\right),C_{\mu }\left(tu\right)\right),\left(\gamma_{r_{\varphi^{\prime }}}\left(tu\right),C_{\gamma }\left(tu\right)\right)/tu\;\epsilon \;E\right\}. \end{array}$$

Where $r_{\varphi^{\prime }}$ is the intuitionistic fuzzy credibility relation relative to φ′, which represents the edge-related intuitionistic fuzzy credibility values.

Definition 2: let T be a universal set. The intuitionistic fuzzy credibility graph on T is a pair G = (V, E). The edge constraints of an intuitionistic fuzzy credibility graph determine the allowable connection of the intuitionistic fuzzy information in regard to two vertices and the related edge. For an edge linking vertices *t* and *u*, the membership degree of the edge cannot exceed the membership degree of either of its endpoints. Thus, the edge membership is restricted by the lowest membership degree of the affected vertices. Similarly, the edge non-membership degree is lower restricted by the maximal non-membership degree of its incident vertices. These requirements ensure that an edge has neither greater membership nor weaker non-membership than allowed by its endpoint information. The accompanying credibility restrictions place similar restrictions on the credibility of membership and non-membership. The edge inequalities guarantee the consistency of the intuitionistic fuzzy credibility information for the representation of the relationships between the vertices. such that


$$\begin{array}{l} \mu_{ij}\left(tu\right)\leq \min\left(\mu_{i}\left(t\right),\;\mu_{j}\left(u\right)\right)\\ \gamma_{ij}\left(tu\right)\;\geq \max\left(\gamma_{i}\left(t\right),\;\gamma_{j}\left(u\right)\right)\\ C_{\mu_{ij}}\;\left(tu\right)\leq min\left(C_{\mu_{i}}\left(t\right),\;C_{\mu_{j}}\left(u\right)\right)\\ C_{\gamma_{ij}}\left(tu\right)\;\geq \max\left(C_{\gamma_{i}}\left(t\right),C_{\gamma_{j}}\left(u\right)\right) \end{array}$$

where, *t, u ϵ V*, and *tu* ϵ *E*. These inequalities are considered the theoretical restrictions of the IFCG definition. In the proposed research-paper framework, the actual edge-weight construction is subsequently defined in Section 5.

### 3.1 Adjacent matrix of IFCG

If $G=\left(\varphi^{\prime },\;r_{\varphi^{\prime }}\right)$ be an IFCG, then the adjacent matrix of G is defined as


$$\begin{array}{l} A\left(G\right)={\left[\left(a,b\right)\right]}_{n\times n} \end{array}$$

Where $a=\left(\mu_{r_{\varphi^{\prime }}}\left(tu\right),C_{\mu }\left(tu\right)\right)$ and $b=\left(\gamma_{r_{\varphi^{\prime }}}\left(tu\right),C_{\gamma }\left(tu\;\right)\right)$.

### 3.2 Laplacian matrix of IFCG

If *G* = (*V, E*) be an IFCG, then the Laplacian matrix of *G* is defined as


$$\begin{array}{l} L\left(G\right)=D\left(G\right)-A\left(G\right) \end{array}$$ (1)

Where *D*(*G*) is the degree matrix of *G* with respect to *A*(*G*).

### 3.3 Node-based Laplacian energy

Node-based Laplacian energy (NBLE) is a structural metric at the node level that assesses the contribution or connection attributes of a specific node, derived from the values in its associated row of the graph Laplacian matrix.

Definition 1: Let *G* = (*V, E*) be an IFCG with Laplacian matrix *L*(*G*) = [*L*_*ij*_]. The node-based Laplacian energy associated with a node *v*_*i*_ ∈ *V* is characterized as the cumulative absolute sum of the respective Laplacian elements in that row. Mathematically it can be defined as


$$\begin{array}{l} E_{i}=\underset{j}{\sum }|L_{ij}|;i,j=1,2,\ldots ,n \end{array}$$ (2)

Where *E*_*i*_ is the node-based Laplacian energy of *i*-th node, and *L*_*ij*_ is the (*i, j*)-th element in Laplacian matrix *L*(*G*).

The suggested *E*_*i*_ is a node-level structural descriptor that is produced by keeping the absolute sum of the rows of each node. We should differentiate it from the traditional graph Laplacian energy, which is commonly described as a graph-level spectral variable based on the eigenvalues of the Laplacian matrix. The current formulation preserves the row-wise values *E*_*i*_ to offer node-specific structural information for the next graph representation learning.

Definition 2: Let *L*(*G*) = [*L*_*ij*_] be the Laplacian matrix of the IFCG, the maximum absolute row sum is defined as


$$\begin{array}{l} \|L{\|}_{\infty }=\;\underset{i}{\max}\underset{j}{\sum }|L_{ij}|=\;\underset{i}{\max}\left(E_{i}\right) \end{array}$$

The maximal absolute row-sum norm of the credibility Laplacian is the largest node-based Laplacian energy of all nodes. Hence, *E*_*i*_ is a node-wise measure of the structure, whereas ||*L*||_∞_ is the maximum over the whole graph.

Definition 3: Let A be the symmetric non-negative adjacency matrix and *L*(*G*) = *D* − *A* be the Laplacian matrix of the IFCG, then the NBLE in terms of the degree of the vertices is defined as


$$\begin{array}{l} E_{i}=\underset{j}{\sum }|L_{ij}|=d_{i}+\underset{j\neq i}{\sum }A_{ij}=2d_{i} \end{array}$$

where $d_{i}=\underset{j\neq i}{\sum }A_{ij}$ is the weighted degree of node *v*_*i*_. Therefore, NBLE offers an explicit node-level Laplacian-based structural descriptor, which is included in the proposed IFCG-GNN architecture.

## 4 Problem statement

The explosion of scientific literature has generated a significant demand for accurate and scalable categorization of research papers to organize academic knowledge and identify acceptable study fields. Traditional text classification approaches mostly focus on document attributes independently, without the ability to leverage structural links across research articles. Graph neural networks (GNNs) may encode relational information, but traditional GNN models often treat nodes with crisp numerical properties, making them less effective at managing the uncertainty, imprecision, and hesitancy included in academic research and domain assignments. In addition, existing graph-based methods tend to treat all nodes equally, without explicitly measuring the structural value of different nodes in the graph. Furthermore, the traditional graph convolution may not be able to properly capture the gradual dispersion of information over the related research papers. These restrictions inspire the creation of a structure-aware and uncertainty-aware graph learning system. Therefore, in this paper, we propose an Intuitionistic Fuzzy Credibility Graph Neural Network (IFCG-GNN) to embed membership, non-membership, and trust information into research-document representations. We present a metric called node-based Laplacian energy (NBLE) to quantify the structural importance of individual nodes with the graph Laplacian. The learned node-energy weights are used to give more structural knowledge in the learning process. In addition, we introduce a diffusion layer to promote the information dissemination among linked research publications. The proposed system jointly uses semantic information, intuitionistic uncertainty, graph structure, node-level relevance, and diffusion-based propagation for classification. The efficacy is evaluated systematically by comparing with the existing baseline models like MLP, GCN, GraphSAGE, GAT, diffusion-convolutional neural networks (DCCN) (Atwood and Towsley, 2016), diffusion jumps-GNN (DJ-GNN) (Begga et al., 2025), and A component-wise comparison is undertaken to evaluate individual contribution of each component of the proposed framework. The overarching goal is to provide a robust and interpretable graph-based classification framework that may improve the representation and classification of research articles when faced with uncertain and relational data.

## 5 Methodology

The proposed system blends Intuitionistic Fuzzy Credibility Graph (IFCG) representation, node-based Laplacian energy (NBLE), diffusion-based feature propagation, and Graph Neural Networks (GNNs) for research-paper categorization. The technique mixes intuitionistic fuzzy information and graph-based deep learning to capture uncertainty and structural links between research publications. Semantic characteristics are initially derived from the research-paper text using Sentence-BERT embeddings and similarity metrics. These traits are converted into intuitionistic fuzzy credibility attributes, which include membership values, non-membership values, and trust values. The four-tuple structure obtained this way gives a fuller depiction of uncertainty than the standard crisp feature representations. Then a graph is built by connecting papers according to their semantic or structural similarities. The graph Laplacian captures the local structural properties and interactions between neighboring nodes. Then, a metric called node-based Laplacian energy (NBLE) is proposed to quantify the structural contribution of each individual node. The energy is produced by calculating a row-wise norm of the respective Laplacian representation and then normalizing to get node-level weights. These weights are added to the node-feature representation before graph convolution, thereby highlighting research papers that are structurally important. Also, we add a diffusion layer to the suggested design to improve information dispersion over the graph. Together with the IFG representation, node-level Laplacian weighting, diffusion mechanism, and GNN, the model can learn uncertainty-aware semantic information and graph-structural relationships. The suggested framework is trained for multi-class research-paper classification. Accuracy, precision, recall, F1-score, confusion matrices, loss, and accuracy curves are used to assess it. We evaluate its performance in comparison to standard baseline models such as MLP, GCN, GraphSAGE, GAT, DCCN, and DJ-GNN under the same experimental scenarios. Importantly, the suggested methodology may utilize the fuzzy uncertainty, the node-level structural relevance, and the diffusion-based graph representation learning in a combined manner, which provides a potentially more informative and robust technique for automated research-paper categorization (see Figure 1).

**Figure 1:**
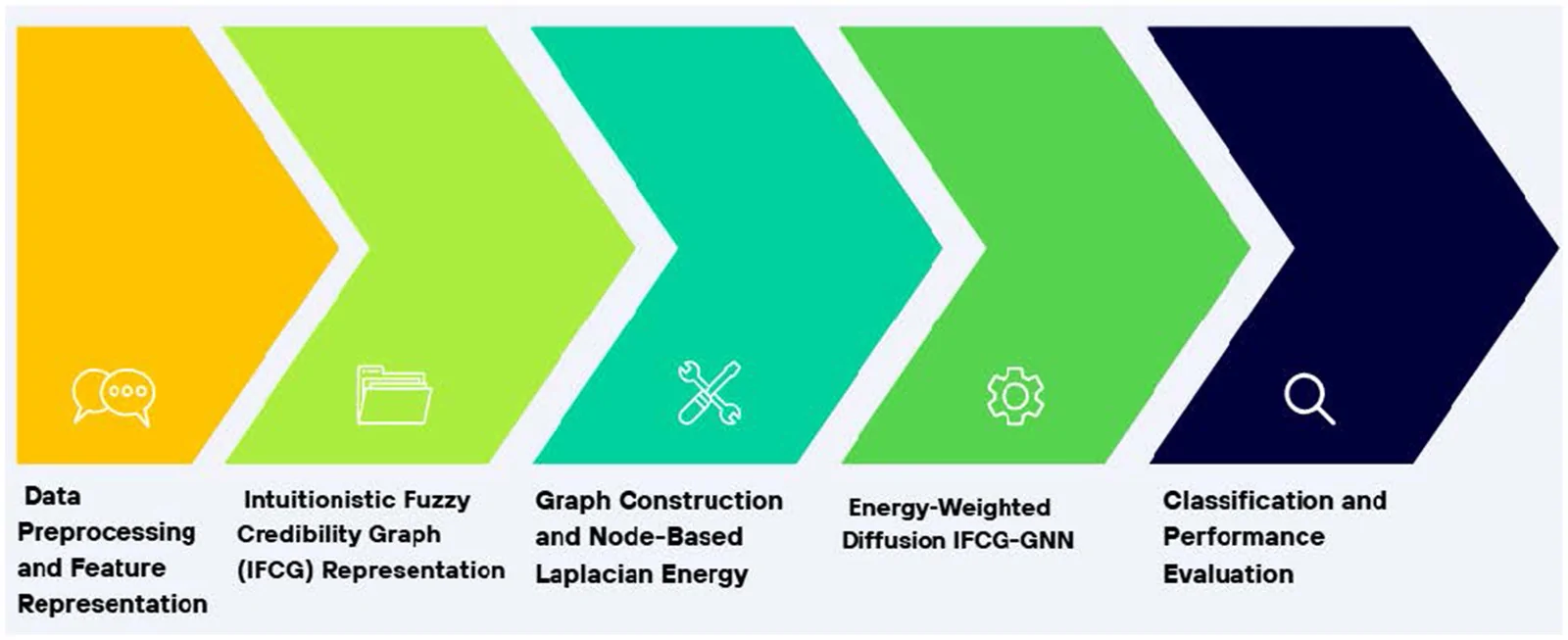
Overall workflow of the proposed IFCG-GNN framework.

### 5.1 About dataset

The research articles used in this work were obtained from the OpenAlex academic database and consist of 12,552 articles with the title, abstract, and keywords. These features of the text are aggregated into a single semantic representation for each document and encoded into a dense numerical embedding using the Sentence-BERT (SBERT) model. The semantic pertinence of each manuscript to the four evaluated fields; computer science, engineering, fuzzy systems, and bio-sciences—is measured by cosine similarity between the paper's embedding and the respective domain representation. Consequently, every document is characterized by four domain-specific similarity metrics, labeled as $S_{i}^{CS}$, $S_{i}^{Eng}$, $S_{i}^{Fuzzy}$, and $S_{i}^{Bio}$. These quantitative semantic attributes later provide the foundation for establishing the intuitionistic membership and non-membership degrees, as well as for developing the IFCG-based node representation. This method systematically converts unstructured academic text into quantitative semantic attributes appropriate for IFCG and GNN-based categorization.

### 5.2 Algorithm

**Input:** Research-paper dataset *V*_*i*_ = {*v*_1_, *v*_2_, …, *v*_*N*_}, where *V*_*i*_ is set of all research papers, and *N* = 12,552.

**Output:** Predicted research-paper class labels

**Step 1:** Preprocessing of data—removal of duplicates and missing values, normalization for numerical features.

**Step 2:** Paper embedding/similarity value is got by Sentence-BERT, and the similarity of research papers is calculated.

**Step 3:** Calculate the intuitionistic fuzzy four-tuple for every research paper which include membership, membership credibility, non-membership and non-membership credibility.

Each research publication is mapped to a node of a graph in the proposed framework. As a research article might be associated with several study domains to varying degrees, the node information is expressed in terms of membership, non-membership, and their respective credibility quantities as follows

Let the domain similarity values for paper *v*_*i*_ be $S_{i}^{CS}$, $S_{i}^{Eng}$, $S_{i}^{Fuzzy}$, and $S_{i}^{Bio}$

The membership degree of paper *v*_*i*_ is defined as


$$\begin{array}{l} \mu_{i}=max\left\{S_{i}^{CS},S_{i}^{Eng},S_{i}^{Fuzzy},S_{i}^{Bio}\;\right\}. \end{array}$$

In this present work, we choose β = 0.5, meaning that half of the non-membership potential is assigned to ν_*i*_ and the rest is the uncertainty component. So, the non-membership degree is defined as


$$\begin{array}{l} \gamma_{i}=\beta \left(1-\mu_{i}\right),\text{Where }\beta =\;0.5 \end{array}$$

The value β = 0.5 provides a balanced compromise between non-membership and the remaining uncertainty component.

The credibility associated with membership and non-membership is defined as


$$\begin{array}{l} C_{\mu_{i}}=\frac{\mu_{i}}{\mu_{i}+\gamma_{i}},\;and\;C_{\gamma_{i}}=\frac{\gamma_{i}}{\mu_{i}+\gamma_{i}} \end{array}$$

The Intuitionistic credibility numbers related to the research paper *v*_*i*_ are represented as follows:


$$\begin{array}{l} Z_{i}=\left(\mu_{i},\gamma_{i},C_{\mu_{i}},C_{\gamma_{i}}\right) \end{array}$$

**Step 4:** For each edge construction between two research paper nodes *v*_*i*_ and *v*_*j*_, we are employing Euclidean distance between the respective node intuitionistic credibility vectors *Z*_*i*_ = (μ_*i*_, γ_*i*_, *C*_μ_*i*__, *C*_γ_*i*__) and *Z*_*j*_ = (μ_*j*_, γ_*j*_, *C*_μ_*j*__, *C*_γ_*j*__) as follows:


$$\begin{array}{l} c_{ij}=\|Z_{i}-Z_{j}{\|}_{2}=\sqrt{\overset{4}{\underset{k=1}{\sum }}{\left(Z_{ik}-Z_{jk}\right)}^{2}} \end{array}$$

The adjacency matrix is computed by using $A\left(G\right)=e^{-C_{ij}}$.

Where *c*_*ij*_ is the Euclidean distance between research articles with their intuitionistic fuzzy Credibility values, $e^{-C_{ij}}$ is the exponential kernel, which is used to transform the non-negative credibility distance between two research-paper nodes into a bounded similarity weight. Since the kernel reduces monotonically with distance, papers with comparable intuitionistic credibility representations are more strongly connected, and dissimilar papers are less strongly connected. This gives a smooth and continuous representation of inter-node interactions for later graph propagation.

**Step 5:** In this phase we are creating degree matrix by using an adjacency matrix. And then we build the Laplacian matrix using Equation 1 as follows:

The weighted degree of node *v*_*i*_ in *A*(*G*) is


$$\begin{array}{l} d_{i}=\overset{n}{\underset{j=1}{\sum }}A_{ij} \end{array}$$

The degree matrix is the diagonal matrix, and it is defined as


$$\begin{array}{l} D=diag\left(d_{1},d_{2},\ldots ,d_{n}\right) \end{array}$$

The Laplacian matrix is defined as


$$\begin{array}{l} L=D-A=\{\begin{array}{c} d_{i}-A_{ii},\;i=j\\ -A_{ij},\;i\neq j\; \end{array} \end{array}$$

**Step 6:** Each node's Laplacian energy is determined using Equation 2, followed using min-max normalization to achieve normalized adaptive weights.


$$\begin{array}{l} \tilde{E_{i}}=\frac{E_{i}-E_{\min}}{E_{\max}-E_{\min}} \end{array}$$

Where, *E*_min_ = *min*(*E*_*i*_), *E*_max_ = *max*(*E*_*i*_).

Thus, the node-energy weight vector is represented as


$$\begin{array}{l} Ẽ={\left[\tilde{E_{1},}\tilde{E_{2}},\ldots ,\tilde{E_{N}}\right]}^{T} \end{array}$$

**Step 7:** In this phase, we are developing the input node feature matrix as outlined below.

Define *f*_*i*_ as the input feature vector, expressed as


$$\begin{array}{l} f_{i}=\left[S_{i}^{CS},S_{i}^{Eng},S_{i}^{Fuzzy},S_{i}^{Bio},\mu_{i},\gamma_{i},C_{\mu_{i}},C_{\gamma_{i}}\right] \end{array}$$

Thereafter, the normalized node based Laplacian energy is included.


$$\begin{array}{l} X_{i}=\left[f_{i},\tilde{E_{i}}\right]=\left[S_{i}^{CS},S_{i}^{Eng},S_{i}^{Fuzzy},S_{i}^{Bio},\mu_{i},\gamma_{i},C_{\mu_{i}},C_{\gamma_{i}},\tilde{E_{i}}\right] \end{array}$$

The variable *f*_*i*_ corresponds to the feature vector associated with paper i, $\tilde{E_{i}}$ is the normalized Laplacian energy based on nodes, *X*_*i*_ is the enhanced initial feature vector, and *N* refers to the number of research papers or nodes.

**Step 8:** In this step, we have transformed the class labels into a coded format and then partitioned the dataset accordingly. Identify the four disciplines as {Bio Sciences, Computer Science, Engineering, Fuzzy}. The encoding of labels is as follows: Bio Sciences is represented by 0, Computer Science by 1, Engineering by 2, and Fuzzy by 3. Following label encoding, the full dataset is segmented into training and testing subsets. In this scenario, two division ratios (80:20 and 70:30) are examined to determine the strength of the suggested model.

**Step 9:** To embed graph-structural information ahead of GNN propagation, Laplacian diffusion is executed as


$$\begin{array}{l} H^{\left(0\right)}=X_{i}-\alpha LX_{i}, \end{array}$$

with *H*^(0)^ representing the input node-feature matrix, *L* indicating the graph Laplacian, and α serving as the coefficient that modulates the strength of propagation. This yields a structurally polished node representation that functions as the preliminary representation for ensuing GNN learning.

*H*^(0)^ denotes the input node-feature matrix, *L* represents the graph Laplacian, α is the diffusion coefficient controlling the propagation strength. This produces a structurally smoothed node representation that serves as the initial representation for subsequent GNN learning.

**Step 10:** The adjacency matrix *A* is used to propagate the diffused representation across the graph. The normalized node Laplacian energy is adopted to adaptively emphasize the significance of structural nodes in the propagation process:


$$\begin{array}{l} H^{\left(l+1\right)}=\sigma \left[\tilde{E_{i}}⊙GCN\left(H^{\left(l\right)},A\right)\right] \end{array}$$

where $\tilde{E_{i}}$ is the node-wise energy instructions, ⊙ is the node-wise multiplication, and σ is the ReLU activation function. Thus, the proposed architecture combines graph diffusion, GNN propagation, and Laplacian energy guidance into a unified representation-learning framework.

**Step 11:** Train the proposed IFCG-GNN with systematic variation of learning rate η and diffusion coefficient α by simultaneous evaluation of three hidden dimensions (*h*) and three training epochs (*e*).

In particular, η, α* ϵ* {0.001, 0.005, 0.01}, *h ϵ* {16, 32, 64}, *e ϵ* { 200, 300, 400}.

For each set of (η, α), the GNN carries out generalized diffusion and graph propagation:


$$\begin{array}{l} H^{\left(l\right)}=H^{\left(l-1\right)}-\alpha LH^{\left(l-\;1\right)},\\ H^{\left(l+1\right)}=\sigma \left[\tilde{E_{i}}⊙GCN\left(H^{\left(l\right)},A\right)\right]\;for\;l=\;1,\ldots ,L. \end{array}$$

After the classification layer, $Z=H^{\left(L\right)}W_{0}+b_{0},\;\hat{y_{i}}=\arg\underset{k}{\max}Z_{ik}\;for\;k=\;4$.

The parameters of the model are adjusted using the Adam algorithm with a learning rate η by minimizing the cross entropy


$$\begin{array}{l} L=-\frac{1}{\left|V_{train}\right|}\underset{i\in V_{train}}{\sum }log\frac{\text{exp}\left(Z_{i,y_{i}}\right)}{\sum_{k=1}^{K}\text{exp}\left(Z_{ik}\right)} \end{array}$$

**Step 12:** The assessment of the model is conducted through accuracy, precision, recall, the confusion matrix, and the F1-score.

## 6 Results and discussion

In this section, we analyze the effectiveness and robustness of the proposed IFCG-GNN framework under various diffusion parameters, learning rates, hidden dimensions, training epochs, and network depths. We tested how diffusion intensity (α), learning rate, and network depth affect the performance of the proposed IFCG-GNN model. Experiments involve varying α, learning rates, number of hidden layers, and epochs while other parameters remain constant. The experiments were carried out with 80:20 and 70:30 training-testing splits with single-layer and double-layer network designs to comprehensively evaluate the effect of these factors. The performance was assessed by calculating the training and test accuracy, loss, precision, recall, macro-F1, and weighted-F1. The experimental results are presented in Tables 2a–d. The abbreviations used in the tables are as follows: Lr: learning rate, HL: hidden layer, Tr_Acc: Train Accuracy, Te_Acc: Test Accuracy, Tr_Loss: Train Loss, Te_Loss: Test Loss, M_Prec: macro precision, M_rec: macro recall, M_F1: macro F1, We_F1: Weighted F1. The graphical analysis of loss, accuracy and confusion matrix for the selected best performing setup was attached, and we discussed complexity and scalability of the proposed model.

**Table 2a:** Single layer network (80:20).

Alpha (α)	Lr	HL	BestEpochs	Performance metrics							
				Tr_Acc	Te_Acc	Tr_Loss	Te_Loss	M_Prec	M_rec	M_F1	We_F1
0.001	0.001	16	400	0.8665	0.8785	0.4453	0.4206	0.8745	0.8714	0.8696	0.8796
		32	400	0.9412	0.9470	0.2545	0.2386	0.9440	0.9498	0.9451	0.9474
		64	400	0.9593	0.9669	0.1928	0.1796	0.9640	0.9693	0.9657	0.9670
	0.005	16	400	0.9835	0.9829	0.0945	0.0866	0.9802	0.9843	0.9819	0.9830
		32	400	0.9902	0.9904	0.0652	0.0602	0.9894	0.9911	0.9902	0.9904
		64	400	0.9937	0.9932	0.0540	0.0494	0.9920	0.9936	0.9928	0.9932
	0.01	16	400	0.9918	0.9920	0.0549	0.0509	0.9911	0.9923	0.9917	0.9920
		32	400	0.9946	0.9940	0.0419	0.0406	0.9936	0.9942	0.9939	0.9940
		64	400	0.9966	0.9964	0.0340	0.0345	0.9961	0.9965	0.9963	0.9964
0.005	0.001	16	400	0.9428	0.9538	0.2100	0.1919	0.9518	0.9560	0.9526	0.9537
		32	400	0.9577	0.9658	0.1689	0.1562	0.9627	0.9681	0.9646	0.9659
		64	400	0.9794	0.9801	0.1125	0.1031	0.9777	0.9811	0.9792	0.9802
	0.005	16	400	0.9885	0.9877	0.0600	0.0569	0.9864	0.9883	0.9873	0.9877
		32	400	0.9934	0.9944	0.0432	0.0412	0.9938	0.9947	0.9942	0.9944
		64	400	0.9949	0.9940	0.0378	0.0388	0.9933	0.9943	0.9938	0.9940
	0.01	16	400	0.9940	0.9936	0.0405	0.0400	0.9928	0.9938	0.9933	0.9936
		32	400	0.9968	0.9944	0.0290	0.0326	0.9940	0.9942	0.9941	0.9944
		64	300	0.9973	0.9948	0.0276	0.0325	0.9943	0.9949	0.9946	0.9948
0.01	0.001	16	400	0.9284	0.9363	0.2256	0.2111	0.9347	0.9391	0.9346	0.9364
		32	400	0.9635	0.9669	0.1416	0.1296	0.9638	0.9689	0.9657	0.9671
		64	400	0.9894	0.9892	0.0748	0.0687	0.9880	0.9898	0.9888	0.9893
	0.005	16	400	0.9896	0.9900	0.0615	0.0566	0.9896	0.9897	0.9896	0.9900
		32	400	0.9928	0.9936	0.0399	0.0378	0.9931	0.9936	0.9933	0.9936
		64	400	0.9966	0.9940	0.0290	0.0320	0.9939	0.9937	0.9938	0.9940
	0.01	16	400	0.9933	0.9928	0.0371	0.0375	0.9924	0.9925	0.9924	0.9928
		32	400	0.9950	0.9948	0.0274	0.0322	0.9946	0.9944	0.9945	0.9948
		64	300	0.9969	0.9944	0.0252	0.0308	0.9941	0.9941	0.9941	0.9944

**Table 2b:** Single layer network (70:30).

Alpha (α)	Lr	HL	BestEpochs	Performance metrics							
				Tr_Acc	Te_Acc	Tr_Loss	Te_Loss	M_Prec	M_rec	M_F1	We_F1
0.001	0.001	16	400	0.9204	0.9270	0.3266	0.3157	0.9247	0.9287	0.9242	0.9273
		32	400	0.9382	0.9387	0.2505	0.2437	0.9358	0.9408	0.9361	0.9391
		64	400	0.9606	0.9623	0.1825	0.1771	0.9588	0.9648	0.9606	0.9626
	0.005	16	400	0.9847	0.9825	0.0989	0.0952	0.9801	0.9837	0.9816	0.9826
		32	400	0.9907	0.9907	0.0722	0.0684	0.9895	0.9914	0.9904	0.9907
		64	400	0.9936	0.9939	0.0534	0.0507	0.9930	0.9944	0.9937	0.9939
	0.01	16	400	0.9913	0.9910	0.0561	0.0521	0.9896	0.9915	0.9905	0.9910
		32	400	0.9956	0.9955	0.0397	0.0387	0.9951	0.9956	0.9954	0.9955
		64	400	0.9958	0.9955	0.0336	0.0333	0.9952	0.9957	0.9954	0.9955
0.005	0.001	16	400	0.9342	0.9341	0.2388	0.2312	0.9308	0.9348	0.9309	0.9347
		32	400	0.9627	0.9634	0.1503	0.1457	0.9606	0.9646	0.9619	0.9635
		64	400	0.9850	0.9822	0.1005	0.0985	0.9801	0.9832	0.9815	0.9823
	0.005	16	400	0.9888	0.9878	0.0590	0.0591	0.9873	0.9878	0.9875	0.9878
		32	400	0.9944	0.9920	0.0417	0.0427	0.9915	0.9922	0.9919	0.9920
		64	400	0.9951	0.9944	0.0318	0.0335	0.9941	0.9945	0.9943	0.9944
	0.01	16	400	0.9920	0.9894	0.0395	0.0405	0.9890	0.9891	0.9890	0.9894
		32	400	0.9953	0.9928	0.0321	0.0357	0.9925	0.9929	0.9927	0.9928
		64	300	0.9970	0.9936	0.0269	0.0309	0.9933	0.9935	0.9934	0.9936
0.01	0.001	16	400	0.9333	0.9344	0.2192	0.2132	0.9309	0.9370	0.9318	0.9353
		32	400	0.9731	0.9713	0.1303	0.1268	0.9687	0.9718	0.9700	0.9714
		64	400	0.9840	0.9854	0.0998	0.0972	0.9844	0.9858	0.9851	0.9854
	0.005	16	400	0.9879	0.9888	0.0557	0.0542	0.9883	0.9892	0.9887	0.9888
		32	400	0.9936	0.9907	0.0392	0.0405	0.9902	0.9906	0.9904	0.9907
		64	300	0.9957	0.9923	0.0363	0.0382	0.9918	0.9922	0.9919	0.9923
	0.01	16	400	0.9939	0.9923	0.0356	0.0378	0.9918	0.9924	0.9921	0.9923
		32	400	0.9964	0.9923	0.0311	0.0341	0.9921	0.9919	0.9920	0.9923
		64	400	0.9977	0.9939	0.0208	0.0289	0.9934	0.9937	0.9936	0.9939

**Table 2c:** Double layer network (80:20).

Alpha (α)	Lr	HL	BestEpochs	Performance metrics							
				Tr_Acc	Te_Acc	Tr_Loss	Te_Loss	M_Prec	M_rec	M_F1	We_F1
0.001	0.001	16	400	0.9761	0.9797	0.1032	0.0939	0.9785	0.9801	0.9792	0.9797
		32	400	0.9882	0.9865	0.0691	0.0650	0.9853	0.9864	0.9858	0.9865
		64	400	0.9946	0.9920	0.0397	0.0420	0.9909	0.9925	0.9917	0.9920
	0.005	16	400	0.9957	0.9936	0.0272	0.0372	0.9935	0.9936	0.9935	0.9936
		32	300	0.9960	0.9944	0.0255	0.0325	0.9939	0.9945	0.9942	0.9944
		64	200	0.9967	0.9944	0.0225	0.0328	0.9941	0.9943	0.9942	0.9944
	0.01	16	300	0.9960	0.9894	0.0257	0.0397	0.9890	0.9890	0.9890	0.9894
		32	200	0.9978	0.9942	0.0205	0.0316	0.9940	0.9939	0.9939	0.9942
		64	200	0.9978	0.9936	0.0150	0.0270	0.9931	0.9937	0.9934	0.9936
0.005	0.001	16	400	0.9491	0.9442	0.1566	0.1498	0.9412	0.9449	0.9427	0.9444
		32	400	0.9859	0.9805	0.0496	0.0628	0.9791	0.9799	0.9795	0.9805
		64	400	0.9911	0.9841	0.0379	0.0478	0.9830	0.9839	0.9834	0.9841
	0.005	16	400	0.9883	0.9853	0.0392	0.0638	0.9843	0.9852	0.9847	0.9853
		32	400	0.9954	0.9849	0.0231	0.0591	0.9838	0.9847	0.9843	0.9849
		64	400	0.9980	0.9940	0.0151	0.0346	0.9937	0.9940	0.9938	0.9940
	0.01	16	400	0.9920	0.9857	0.0308	0.0522	0.9849	0.9856	0.9852	0.9857
		32	400	0.9966	0.9908	0.0173	0.0500	0.9901	0.9905	0.9903	0.9908
		64	300	0.9983	0.9932	0.0104	0.0494	0.9930	0.9931	0.9930	0.9932
0.01	0.001	16	400	0.9359	0.9375	0.4259	0.4572	0.9350	0.9332	0.9340	0.9373
		32	400	0.9834	0.9777	0.0720	0.1232	0.9761	0.9777	0.9768	0.9777
		64	400	0.9929	0.9849	0.0224	0.0855	0.9843	0.9849	0.9846	0.9849
	0.005	16	400	0.9879	0.9888	0.0557	0.0542	0.9883	0.9892	0.9887	0.9888
		32	400	0.9936	0.9907	0.0392	0.0405	0.9902	0.9906	0.9904	0.9907
		64	300	0.9957	0.9923	0.0363	0.0382	0.9918	0.9922	0.9919	0.9923
	0.01	16	400	0.9869	0.9769	0.0438	0.0922	0.9761	0.9752	0.9756	0.9769
		32	400	0.9969	0.9849	0.0154	0.0795	0.9843	0.9844	0.9844	0.9849
		64	400	0.9983	0.9892	0.0047	0.1090	0.9889	0.9888	0.9889	0.9892

**Table 2d:** Double layer network (70:30).

Alpha (α)	Lr	HL	BestEpochs	Performance metrics							
				Tr_Acc	Te_Acc	Tr_Loss	Te_Loss	M_Prec	M_rec	M_F1	We_F1
0.001	0.001	16	400	0.9754	0.9734	0.1003	0.0987	0.9715	0.9724	0.9719	0.9735
		32	400	0.9901	0.9888	0.0558	0.0579	0.9882	0.9889	0.9885	0.9888
		64	400	0.9940	0.9915	0.0415	0.0447	0.9914	0.9913	0.9914	0.9915
	0.005	16	400	0.9954	0.9939	0.0277	0.0357	0.9934	0.9934	0.9934	0.9939
		32	400	0.9974	0.9928	0.0181	0.0313	0.9925	0.9925	0.9925	0.9928
		64	400	0.9982	0.9950	0.0122	0.0277	0.9947	0.9946	0.9947	0.9950
	0.01	16	400	0.9972	0.9902	0.0211	0.0407	0.9899	0.9899	0.9899	0.9902
		32	200	0.9978	0.9942	0.0205	0.0316	0.9940	0.9939	0.9939	0.9942
		64	200	0.9978	0.9936	0.0150	0.0270	0.9931	0.9937	0.9934	0.9936
0.005	0.001	16	400	0.9380	0.9379	0.1615	0.1647	0.9345	0.9348	0.9346	0.9379
		32	400	0.9820	0.9780	0.0629	0.0719	0.9774	0.9766	0.9770	0.9780
		64	400	0.9915	0.9854	0.0388	0.0503	0.9844	0.9851	0.9847	0.9854
	0.005	16	400	0.9833	0.9750	0.0506	0.0589	0.9736	0.9741	0.9738	0.9751
		32	400	0.9953	0.9865	0.0223	0.0399	0.9859	0.9856	0.9858	0.9865
		64	400	0.9973	0.9899	0.0129	0.0438	0.9894	0.9901	0.9897	0.9899
	0.01	16	400	0.9885	0.9877	0.0600	0.0569	0.9864	0.9883	0.9873	0.9877
		32	400	0.9934	0.9944	0.0432	0.0412	0.9938	0.9947	0.9942	0.9944
		64	400	0.9949	0.9940	0.0378	0.0388	0.9933	0.9943	0.9938	0.9940
0.01	0.001	16	400	0.9262	0.9233	0.4142	0.4506	0.9185	0.9196	0.9189	0.9235
		32	400	0.9723	0.9684	0.1066	0.1756	0.9673	0.9671	0.9672	0.9684
		64	400	0.9940	0.9806	0.0248	0.1091	0.9795	0.9796	0.9795	0.9806
	0.005	16	400	0.9847	0.9811	0.0537	0.1043	0.9805	0.9805	0.9805	0.9812
		32	400	0.9959	0.9859	0.0159	0.0910	0.9856	0.9852	0.9854	0.9859
		64	400	0.9985	0.9873	0.0071	0.1189	0.9868	0.9870	0.9869	0.9873
	0.01	16	400	0.9528	0.9511	0.1221	0.1401	0.9485	0.9535	0.9501	0.9514
		32	400	0.9972	0.9859	0.0152	0.1017	0.9860	0.9852	0.9856	0.9859
		64	400	0.9987	0.9920	0.0051	0.1251	0.9918	0.9917	0.9918	0.9920

### 6.1 Impact of α

The results we obtained show that the optimal diffusion coefficient relies on the network depth and train-test split. The best weighted F1 of 0.9964 is achieved for the single-layer 80:20 configuration (Table 2a) with α = 0.001, learning rate = 0.01, and hidden dimension 64. For the single-layer 70:30 arrangement (Table 2b), the best weighted F1 of 0.9955 is obtained with the lower diffusion coefficient alpha (α = 0.001). For the double-layer design, it achieves the best overall results in both partitions, obtaining weighted-F1 values of 0.9944, 0.9950 in Tables 2c, d, respectively. These results illustrate that a stronger diffusion does not guarantee a better classification performance. A higher value may over-smooth the features, whereas a smaller value can save more original node-specific information. The results thereby Favor adaptive hyperparameter selection depending on the network depth and graph architecture.

### 6.2 Influence of learning rate

The learning rate controls the speed of updating the model parameters during optimization. Increasing the learning rate from 0.001 to 0.005 and 0.01 usually speeds up convergence, reducing training loss significantly for the bigger hidden dimensions, as seen in Tables 2a–d. The single-layer 80:20 results with alpha = 0.01 indicate that the configuration with learning rate 0.01 and hidden dimension 32 achieves a test accuracy of 0.9948, compared to 0.9671 for the 0.001 learning-rate configuration. Similar gains are seen with a 70:30 split where the configuration of α = 0.001, learning-rate = 0.01, and hidden-dimension = 32 gives about 0.9955 test accuracy. However, the increase in the learning rate should not be considered uniformly beneficial, as various configurations reveal small changes between training and testing loss and accuracy. Thus, the learning rate was evaluated jointly together with alpha and the hidden dimension rather than singly. Our investigations indicate that the choice of learning rate influences both the convergence speed and the generalization, with a learning rate of 0.01 in the top-performing configurations yielding very competitive results; however, the ultimate choice is made based on the testing performance and not only on the training accuracy.

### 6.3 Impact of hidden dimension and epochs

The learning efficiency and convergence of the proposed IFCG-GNN model are affected by the hidden dimensions of 32 and 64, which usually perform and train epochs together. In all four experimental setups, the hidden dimensions of 32 and 64 usually perform better than 16, suggesting that a considerably larger representation space helps capture complicated graph and intuitionistic-fuzzy interactions. However, the increase of the hidden dimension to 64 does not always enhance the performance, especially for the single-layer network 70:30 split, where the best overall result is obtained with 32 hidden units. The ideal number of epochs also depends on the setup. The best models converged around 300 or 400 epochs and did not require the maximum number of epochs in every case. For instance, the best single-layer 80:20 configuration obtains a weighted-F1 of 0.9964 after 400 epochs, whereas the best single-layer 70:30 configuration achieves 0.9955 at 400 epochs. The double-layer models reach their best performance around 300 to 400 epochs with weighted-F1 values of approximately 0.9944 and 0.9950 in 80:20, 70:30 splits, respectively. In general, the results indicate that a satisfactory compromise between representational capacity, convergence, and computational cost may be achieved with 32 hidden dimensions and about 300–400 epochs.

### 6.4 The effect of network depth

Experimental results show a small positive impact of the network depth on the classification performance of the proposed IFCG-GNN framework. The single-layer network performs marginally better than the double-layer network on both 80:20 and 70:30 data divisions. The best single-layer design obtains a weighted F1 of 0.9964 for the 80:20 split and 0.9955 for the 70:30 split, whereas the corresponding best double-layer configurations earn 0.9944 for the 80:20 split and 0.9950 for the 70:30 split. This implies that one graph-convolution layer is enough to capture the local structural relationships in the created graph. The extra layer could cause heavy neighborhood aggregation and feature smoothing, leading to some node-specific discriminative information loss. Therefore, the single-layer design can achieve a better trade-off between prediction performance, model simplicity, and computing efficiency with the current dataset and graph structure.

### 6.5 Graphical representation of best configurations

The numerical findings presented in Tables 2a–d were further validated by graphical analyses utilizing the highest-performing configuration obtained from each testing setting. The convergence is studied using the training/testing loss curves and the generalization with the accuracy curves, respectively, while the confusion matrices provide a class-wise evaluation of the final predictions. The four configurations used are the single-layer 80:20, single-layer 70:30, double-layer 80:20, and double-layer 70:30 networks. The numerical performance presented in the tables is supported by the visual evidence of consistent convergence of the loss curves, high and tightly aligned training/testing accuracy, and dominating diagonal elements in the confusion matrices.

#### 6.5.1 Single layer 80:20 split

See Figure 2 for single layer 80:20 split.

**Figure 2:**
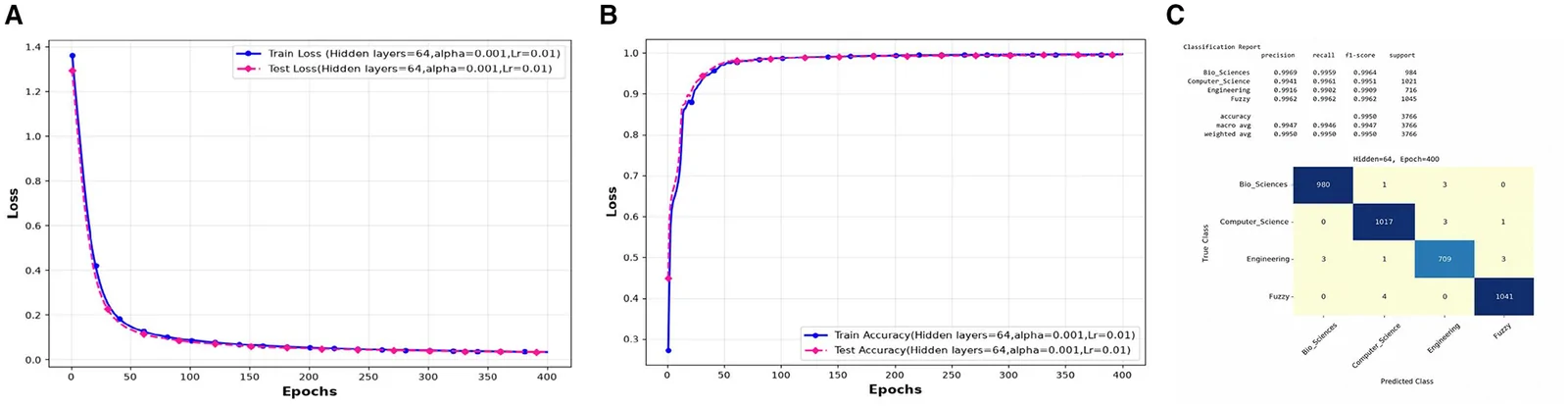
**(A)** Train and test loss curves. **(B)** Train and test accuracy curves. **(C)** Confusion matrix.

#### 6.5.2 Single layer 70:30 split

See Figure 3 for single layer 70:30 split.

**Figure 3:**
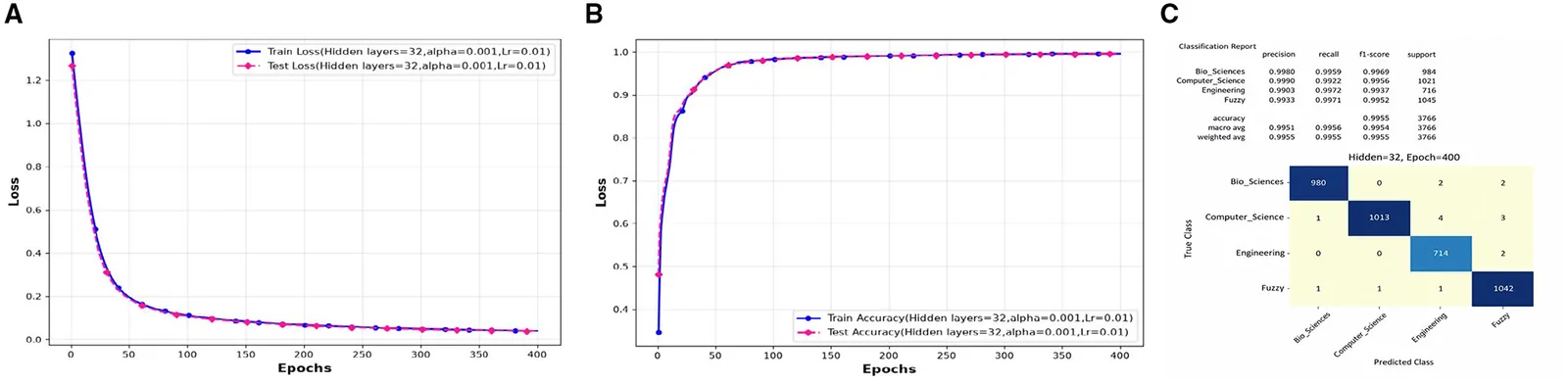
**(A)** Train and test loss curves. **(B)** Train and test accuracy curves. **(C)** Confusion matrix.

#### 6.5.3 Double layer 80:20 split

See Figure 4 for double layer 80:20 split.

**Figure 4:**
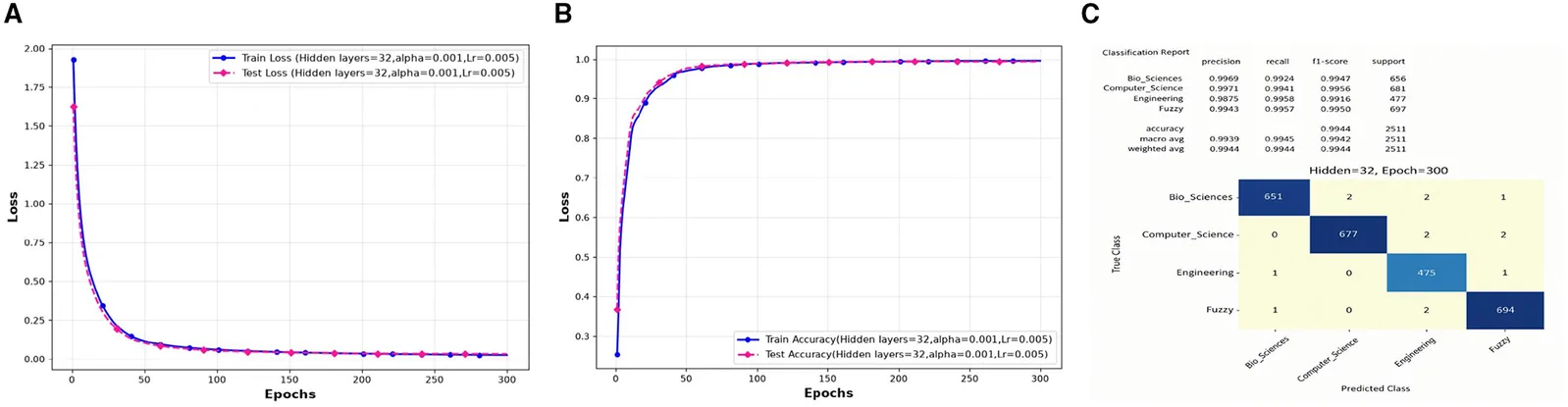
**(A)** Train and test loss curves. **(B)** Train and test accuracy curves. **(C)** Confusion matrix.

#### 6.5.4 Double layer 70:30 split

See Figure 5 for double layer 70:30 split.

**Figure 5:**
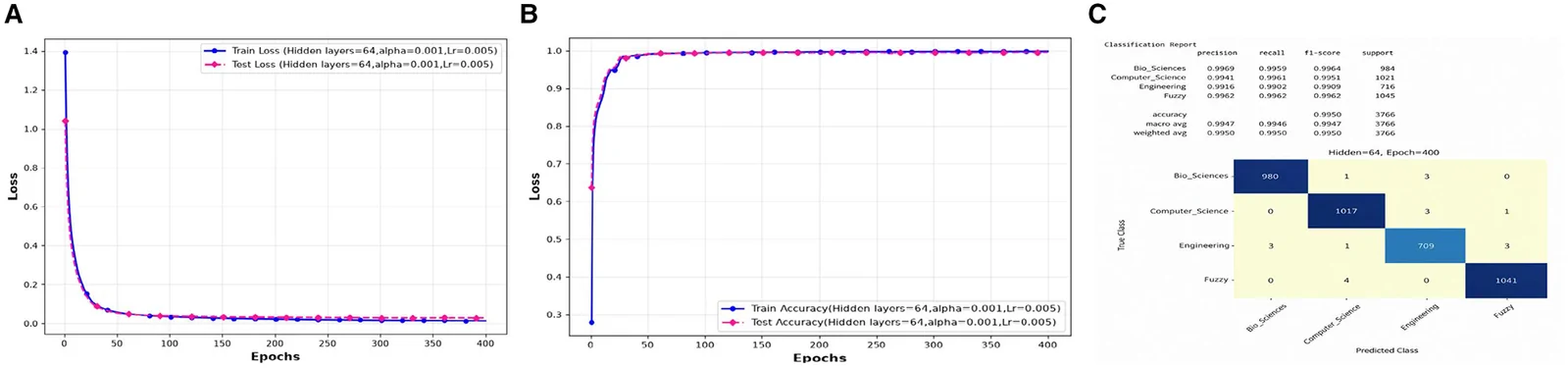
**(A)** Train and test loss curves. **(B)** Train and test accuracy curves. **(C)** Confusion matrix.

### 6.6 Comparative analysis

For a fair comparison, the proposed IFCG-GNN with Node-Based Laplacian Energy and diffusion should be compared with existing baseline models, including MLP, GCN, GraphSAGE, GAT, Diffusion-convolutional neural networks (DCCN) (Atwood and Towsley, 2016), and diffusion-jump GNNs (DJ-GNN) (Begga et al., 2025) using the same dataset, feature representation, train–test partitions, and fixed learning rate = 0.01. The comparison results are in Tables 3a, b for 80:20 split and 70:30 splits, respectively.

**Table 3a:** 80:20 split.

Lr	HL	Models							
		MLP	GCN	GRAPH SAGE	GAT	DCCN	DJ-GNN	Proposed (IFCG-GNN)	
								Single layer (α = 0.001)	Double layer (α = 0.001)
0.01	16	0.9931	0.9734	0.9778	0.9714	0.9762	0.9758	0.9920	0.9894
	32	0.9960	0.9730	0.9859	0.9714	0.9766	0.9766	0.9940	0.9942
	64	0.9956	0.9750	0.9875	0.9722	0.9782	0.9790	0.9964	0.9936

**Table 3b:** 70:30 split.

Lr	HL	Models							
		MLP	GCN	GRAPH SAGE	GAT	DCCN	DJ-GNN	Proposed (IFCG-GNN)	
								Single layer (α = 0.001)	Double layer (α = 0.001)
0.01	16	0.9949	0.9758	0.9807	0.9723	0.9751	0.9755	0.9910	0.9902
	32	0.9949	0.9766	0.9847	0.9737	0.9753	0.9750	0.9955	0.9942
	64	0.9954	0.9758	0.9874	0.9747	0.9750	0.9769	0.9955	0.9936

The experimental results indicate that the proposed IFCG-GNN achieves very competitive classification performance on the train-test split. For the 70:30 division, the single-layer IFCG-GNN obtains the highest accuracy of 0.9955, slightly better than MLP (0.9954) and greatly better than GCN (0.9766), GraphSAGE (0.9874), GAT (0.9747), DCCN (0.9753), and DJ-GNN (0.9769). In the 80:20 split, the single-layer IFCG-GNN obtains the best overall accuracy of 0.9964, better than MLP (0.9960), GCN (0.9762), GraphSAGE (0.9875), GAT (0.9726), DCCN (0.9782), and DJ-GNN (0.9790). Moreover, the double-layer IFCG-GNN is very efficient, with an accuracy of 0.9950 on the 70:30 division and 0.9944 on the 80:20 division. The results show, in general, the efficacy of integrating graph-based learning with intuitionistic fuzzy credibility data for classifying research papers. The best-performing model is the single-layer IFCG-GNN architecture with an 80:20 data partition, learning rate 0.01 and 64 hidden units.

### 6.7 Reproducibility and robustness

In the previous section, 6.6 comparative analysis, we examined different hidden dimensions, network depths, and train-test splits with fixed learning rate = 0.01. These results motivated the robustness evaluation, carried out with a fixed single-layer IFCG-GNN configuration with a learning rate of 0.01, 64 hidden dimensions, alpha=0.001, 400 epochs, and an 80:20 split. The proposed and compared models were evaluated independently with 5 random seeds (42, 52, 62, 72, 82). All runs used the same assessment process for consistency. For each run, accuracy, precision, recall, and F1-score were recorded. Results are reported in Table 4 as mean ± standard deviation over five runs. The repeated-run evaluation offers a stable estimate of the performance variation and repeatability for diverse random initializations.

**Table 4:** Mean ± standard deviation over five separate runs.

Model	Accuracy (%)	Precision (%)	Recall (%)	F1-score (%)
MLP	99.5002 ± 0.1294	99.4939 ± 0.1595	99.4842 ± 0.1182	99.4880 ± 0.1341
GCN	97.5252 ± 0.0928	97.4238 ± 0.1131	97.5648 ± 0.1049	97.4905 ± 0.1071
GRAPHSAGE	98.6457 ± 0.0221	98.6145 ± 0.0431	98.6106 ± 0.0345	98.6113 ± 0.0286
GAT	96.9690 ± 0.1043	96.7842 ± 0.1277	97.0799 ± 0.1284	96.9146 ± 0.1214
DCCN	97.7751 ± 0.0826	97.6067 ± 0.0940	97.8723 ± 0.0738	97.7288 ± 0.0849
DJ-GNN	97.6300 ± 0.2233	97.4555 ± 0.2556	97.7098 ± 0.1987	97.5703 ± 0.2315
Proposed (IFCG-GNN)	99.4743 ± 0.1391	99.4003 ± 0.1781	99.5134 ± 0.1286	99.4539 ±0.1554

As shown in Table 4, the proposed model was able to achieve 99.4743% ± 0.1391% test accuracy, 99.4003% ± 0.1781% precision, 99.5134% ± 0.1286% recall, and 99.4539% ± 0.1554% F1-score. The standard deviations are rather minimal, which implies a consistent run-to-run performance for various random initializations. To make a fair comparison, MLP, GCN, GraphSAGE, GAT, DCNN, and DJ-GNN are likewise tested with the same five-run process and experimental conditions. The means presented are the average prediction performance over the five runs, and the standard deviations are the variance among runs. Therefore, the repeated-run assessment provides evidence of the consistency and repeatability of the observed performance. The statistical significance was determined in the following study using the statistical tests presented independently.

### 6.8 Statistical validation

Table 5 presents the statistical analysis of comparative classification results of the proposed IFCG-GNN with the selected baseline models. To test the statistical significance of the difference in accuracy, the Wilcoxon signed-rank test was used. The *p*-value of IFCG-GNN and MLP is 0.8125, and the effect size is 0.2, which means there is no statistically significant difference at the 5% significance level. When compared with GCN, GraphSAGE, GAT, and DCNN, the *p*-values were also 0.0625, and the Holm-adjusted *p*-values were 0.3750. Comparison with DJ-GNN also gave a *p*-value of 0.0625 and a Holm-adjusted value of 0.3750. Holm-adjusted *p*-values are all greater than 0.05, indicating that none of the observed differences is statistically significant under the adopted significance criterion. Thus, the results show the statistical equivalency of the classification performance of the comparative models and IFCG-GNN in the dataset analyzed.

**Table 5:** The statistical significance of the comparative results.

Comparison	Metric	Test	*W*	*p*-value	Effect size	Holm-adjusted	Significant (α = 0.05)
IFCG-GNN vs. MLP	Accuracy	Wilcoxon signed rank	6.0	0.8125	0.2	0.8125	False
IFCG-GNN vs. GCN	Accuracy	Wilcoxon signed rank	0.0	0.0625	1.0	0.3750	False
IFCG-GNN vsGraphSAGE	Accuracy	Wilcoxon signed rank	0.0	0.0625	1.0	0.3750	False
IFCG-GNN vs. GAT	Accuracy	Wilcoxon signed rank	0.0	0.0625	1.0	0.3750	False
IFCG-GNN vs. DCCN	Accuracy	Wilcoxon signed rank	0.0	0.0625	1.0	0.3750	False
IFCG-GNN vs. DJ-GNN	Accuracy	Wilcoxon signed rank	0.0	0.0625	1.0	0.3750	False

### 6.9 Data leakage prevention and overfitting assessment

The tests were performed in a transductive scenario where the graph, the Laplacian matrix, and the NBLE values were built with all the nodes before the train-test partition. However, the class labels of the test node were not employed in model training, parameter optimization, model selection, and hyperparameter tuning. The training loss was estimated just on the training labels, and the test set was used for final performance evaluation. The test set performance was also not utilized to choose the number of training epochs or the final model parameters. The risk of overfitting was studied by monitoring the training loss and training accuracy during the learning process, as well as by testing the model in five distinct runs with various random initializations. The mean and standard deviation of the test measures were analyzed to evaluate the run-to-run consistency. We also analyzed the difference between training and test performance to identify big performance gaps indicating overfitting. The test set was utilized just for the final evaluation and not for any model-selection considerations.

### 6.10 Ablation study

To evaluate the contributions of the proposed IFCG-GNN components, we conducted a component-wise ablation investigation under a fixed experimental setup. We ablated the diffusion component and the NBLE component separately and together, with all other parameters remaining the same. The studies employed a learning rate of 0.01, a hidden dimension of 64, a diffusion coefficient of α = 0.001, and 400 training epochs. Results are shown in Table 6.

**Table 6:** Component wise ablation study of proposed IFCG-GNN.

Model	Diffusion	IFCG	NBLE weight	NBLE feature	Accuracy (%)	Precision (%)	Recall (%)	F1-score (%)	Training time (s)
Full IFCG-GNN	Yes	Yes	Yes	Yes	99.602	99.604	99.602	99.602	19.94
NBLE	Yes	Yes	No	Yes	99.60	99.60	99.60	99.60	6.32
Diffusion	No	Yes	Yes	Yes	98.29	98.32	98.29	98.29	27.75
Diffusion + NBLE	No	Yes	No	Yes	98.53	98.53	98.53	98.53	6.00

As shown in Table 6, the full IFCG-GNN achieves 99.602% accuracy, while removing diffusion reduces accuracy to 98.288%, which shows the substantial contribution of diffusion to the classification performance. By removing NBLE as model weight, performance is almost unaffected (99.602%), showing a small impact in the current configuration. Removing both components achieves 98.526% accuracy, further supporting the contribution of diffusion-based propagation. We evaluate the contribution of the IFCG component by comparing it to standard graph-learning and diffusion-based baselines such as GCN, GraphSAGE, GAT, DCNN, and DJ-GNN, which do not leverage the proposed intuitionistic credibility graph format. These comparisons offer comparative proof of the use of the suggested IFCG-based graph format inside the assessed experimental environment. In the absence of a specific controlled ablation of IFCG, these results are considered comparative evidence rather than a standalone causal evaluation of the IFCG component.

### 6.11 Complexity and scalability

The computational complexity of IFCG-GNN largely lies in graph creation, node-based Laplacian energy computation, diffusion propagation, and GNN training. The theoretical analysis demonstrates the pairwise distance computation to be [O (*n*^{^2}*d*)], while the practical runtime and memory results in Table 6 give complementary proof of computational viability. Scalability tests were done with a fixed configuration of learning rate (=0.01), diffusion coefficient (α = 0.001), hidden dimension (=64), 400 epochs, and an 80:20 train–test split. This fixed configuration allows for the consistent evaluation of the impact of increasing the size of the graph on the execution time and memory usage by employing ever bigger subsets of the dataset. The theoretical analysis and the empirical measurements reported in Table 7 provide a clear estimate of the computational complexity and scalability properties of the proposed IFCG-GNN framework.

**Table 7:** Runtime and memory-based complexity and scalability analysis of IFCG-GNN.

Dataset size	Feature construction (s)	Graph construction (s)	NBLE computation (s)	Edge construction (s)	Diffusion (s)	GNN training (s)	Total time (s)	Peak memory (MB)
1,000	0.0441	0.0422	0.0275	0.0450	0.0668	6.6069	7.7627	38.1997
2,500	0.0095	0.2838	0.1774	0.1593	0.0062	10.1700	10.9329	238.5401
5,000	0.0085	1.1723	0.7997	0.3774	0.0238	18.8907	21.5091	953.9098
10,000	0.0054	3.0474	1.3526	0.4769	0.0269	12.0850	17.1477	3,815.1616
12,552	0.0019	1.9109	1.6090	0.4932	0.0484	17.3385	21.6069	6,010.7401

## 7 Conclusion

In this work, we proposed a formal Intuitionistic Credibility Fuzzy Graph (ICFG) and an IFCG-GNN for research-paper classification by merging intuitionistic uncertainty representation, graph building, NBLE, and diffusion-based feature propagation. The trials were performed in a transductive scenario. The graph, Laplacian, and NBLE were built before the 80:20 and 70:30 train-test splits, and the test labels were only used during the final assessment. For a more comprehensive evaluation, we compare our technique with traditional and diffusion-based approaches, such as MLP, GCN, GraphSAGE, GAT, DCNN, and DJ-GNN. The single-layer IFCG-GNN with the 80:20 split had the greatest single-run performance, with alpha = 0.001, learning rate = 0.01, hidden dimension = 64, and 400 epochs, with 99.64% accuracy, 99.63% macro-F1, and 99.64% weighted-F1. The suggested model attained an average accuracy of 99.4743 ± 0.1391 over five separate runs, exhibiting consistent classification performance. The corresponding mean macro-F1 was 99.4539 ±0.1554, further confirming the stability of the suggested framework. Ablation studies reveal that while diffusion has a larger observable influence, NBLE mainly offers an explicit node-level Laplacian-energy-based structural representation and weighting, rather than an independently quantifiable increase in forecasting accuracy. In future work, the proposed framework will be extended to regression-oriented prediction tasks and dynamic, diverse research-paper networks to better model complex and evolving connections across academic institutions. More validation will be performed using larger real-world datasets, including OpenAlex subgroups with different sizes and domain classifications, and one or more additional publicly available datasets to evaluate the generalizability and robustness of the proposed methodology. The exploration of more sophisticated GNN architectures will also be done for better prediction efficacy. We will also integrate interpretable GNN approaches with uncertainty-aware prediction systems to improve the transparency, credibility, and real-world relevance of the models.

## Data Availability

The raw data supporting the conclusions of this article will be made available by the authors, without undue reservation.
