# Supplementary material for: An energy-aware diffusion graph neural network based on intuitionistic fuzzy credibility graphs for research-paper classification
Source: Front Artif Intell. 2026 Oct 23;9:1973276. doi: 10.3389/frai.2026.1973276 (PMC13739706; doi:10.3389/frai.2026.1973276)
Supplement: Supplementary file 1 [file Data_Sheet_1.pdf]

Table-1: Single layer 80:20

alpha (α)	Lr	HL	Epochs	Performance metrics							
				**Tr_Acc**	**Te_Acc**	**Tr_Loss**	**Te_Loss**	**M_Prec**	**M_rec**	**M_F1**	**We_F1**
**0.001**	**0.001**	**16**	200	0.7215	0.7368	0.8222	0.7972	0.7338	0.7047	0.7011	0.7291
			300	0.8162	0.8276	0.5917	0.5653	0.8221	0.8109	0.8112	0.8274
		**32**	200	0.8907	0.9052	0.5108	0.4852	0.9069	0.9092	0.9032	0.9058
			300	0.9245	0.9311	0.3313	0.3121	0.9287	0.9344	0.9287	0.9317
		**64**	200	0.9122	0.9192	0.3476	0.3269	0.9172	0.9206	0.9159	0.9197
			300	0.9443	0.9490	0.2439	0.2282	0.9460	0.9517	0.9472	0.9492
	**0.005**	**16**	200	0.9658	0.9681	0.1659	0.1542	0.9646	0.9708	0.9666	0.9685
			300	0.9778	0.9781	0.1194	0.1101	0.9748	0.9799	0.9768	0.9783
		**32**	200	0.9789	0.9809	0.1152	0.1049	0.9785	0.9826	0.9802	0.9809
			300	0.9867	0.9873	0.0821	0.0749	0.9857	0.9884	0.9869	0.9873
		**64**	200	0.9856	0.9869	0.0918	0.0834	0.9847	0.9877	0.9861	0.9869
			300	0.9906	0.9912	0.0672	0.0610	0.9897	0.9918	0.9906	0.9913
	**0.01**	**16**	200	0.9868	0.9841	0.0969	0.0887	0.9827	0.9850	0.9837	0.9841
			300	0.9919	0.9904	0.0689	0.0630	0.9894	0.9912	0.9903	0.9904
		**32**	200	0.9900	0.9904	0.0701	0.0638	0.9888	0.9909	0.9898	0.9905
			300	0.9927	0.9924	0.0517	0.0483	0.9914	0.9927	0.9920	0.9924
		**64**	200	0.9937	0.9924	0.0540	0.0502	0.9913	0.9928	0.9920	0.9925
			300	0.9960	0.9948	0.0410	0.0395	0.9942	0.9950	0.9946	0.9948
**0.005**	**0.001**	**16**	200	0.8899	0.8988	0.3853	0.3602	0.8991	0.8970	0.8947	0.8989
			300	0.9262	0.9367	0.2761	0.2532	0.9341	0.9375	0.9341	0.9366
		**32**	200	0.9125	0.9271	0.3107	0.2878	0.9248	0.9296	0.9240	0.9279
			300	0.9431	0.9522	0.2160	0.1995	0.9491	0.9552	0.9506	0.9524
		**64**	200	0.9463	0.9562	0.2081	0.1923	0.9526	0.9584	0.9542	0.9566
			300	0.9693	0.9721	0.1449	0.1334	0.9690	0.9737	0.9708	0.9723
	**0.005**	**16**	200	0.9781	0.9793	0.1032	0.0967	0.9769	0.9807	0.9785	0.9794
			300	0.9859	0.9841	0.0741	0.0695	0.9824	0.9847	0.9835	0.9841
		**32**	200	0.9891	0.9908	0.0691	0.0632	0.9899	0.9914	0.9906	0.9908
			300	0.9922	0.9924	0.0523	0.0484	0.9918	0.9926	0.9921	0.9924
		**64**	200	0.9896	0.9912	0.0622	0.0587	0.9903	0.9916	0.9909	0.9912
			300	0.9929	0.9928	0.0465	0.0456	0.9922	0.9930	0.9926	0.9928
	**0.01**	**16**	200	0.9899	0.9908	0.0638	0.0598	0.9900	0.9910	0.9905	0.9908
			300	0.9929	0.9928	0.0489	0.0468	0.9919	0.9929	0.9924	0.9928
		**32**	200	0.9933	0.9940	0.0454	0.0432	0.9935	0.9940	0.9937	0.9940
			300	0.9953	0.9936	0.0348	0.0358	0.9931	0.9936	0.9933	0.9936
		**64**	200	0.9962	0.9928	0.0355	0.0370	0.9922	0.9929	0.9926	0.9928
			400	0.9978	0.9944	0.0234	0.0310	0.9941	0.9944	0.9942	0.9944
**0.01**	**0.001**	**16**	200	0.8778	0.8933	0.3823	0.3569	0.8961	0.8902	0.8895	0.8937
			300	0.9082	0.9188	0.2858	0.2664	0.9190	0.9205	0.9170	0.9190
		**32**	200	0.9260	0.9419	0.2562	0.2361	0.9396	0.9447	0.9406	0.9420
			300	0.9501	0.9618	0.1825	0.1676	0.9588	0.9638	0.9605	0.9619
		**64**	200	0.9756	0.9789	0.1309	0.1198	0.9762	0.9805	0.9780	0.9790
			300	0.9847	0.9857	0.0945	0.0862	0.9834	0.9866	0.9848	0.9857
	**0.005**	**16**	200	0.9767	0.9777	0.1018	0.0936	0.9755	0.9778	0.9765	0.9778
			300	0.9867	0.9885	0.0745	0.0684	0.9876	0.9884	0.9880	0.9885
		**32**	200	0.9891	0.9885	0.0646	0.0581	0.9874	0.9886	0.9879	0.9885
			300	0.9917	0.9924	0.0485	0.0445	0.9918	0.9925	0.9922	0.9924
		**64**	200	0.9928	0.9924	0.0461	0.0440	0.9917	0.9924	0.9920	0.9924
			300	0.9954	0.9928	0.0351	0.0358	0.9922	0.9927	0.9924	0.9928
	**0.01**	**16**	200	0.9880	0.9916	0.0604	0.0545	0.9913	0.9913	0.9913	0.9916
			300	0.9917	0.9920	0.0446	0.0426	0.9915	0.9918	0.9916	0.9920
		**32**	200	0.9926	0.9924	0.0422	0.0413	0.9918	0.9926	0.9922	0.9924
			300	0.9941	0.9928	0.0329	0.0355	0.9923	0.9926	0.9924	0.9928
		**64**	200	0.9954	0.9936	0.0319	0.0349	0.9932	0.9935	0.9933	0.9936
			400	0.9973	0.9940	0.0214	0.0294	0.9937	0.9937	0.9937	0.9940

Table-2: Single layer 70:30

alpha (α)	Lr	HL	Epochs	Tr_Acc	Te_Acc	Tr_Loss	Te_Loss	M_Prec	M_rec	M_F1	We_F1
0.001	0.001	16	200	0.8313	0.8463	0.6508	0.6355	0.8606	0.8371	0.8395	0.8462
			300	0.8935	0.9004	0.4290	0.4152	0.9007	0.8988	0.8963	0.9008
		32	200	0.8772	0.8797	0.4862	0.4731	0.8802	0.8754	0.8737	0.8804
			300	0.9163	0.9185	0.3264	0.3172	0.9161	0.9194	0.9148	0.9192
		64	200	0.9154	0.9142	0.3278	0.3172	0.9118	0.9163	0.9106	0.9153
			300	0.9420	0.9426	0.2313	0.2243	0.9391	0.9447	0.9401	0.9432
	0.005	16	200	0.9653	0.9679	0.1703	0.1648	0.9646	0.9699	0.9664	0.9681
			300	0.9776	0.9766	0.1236	0.1194	0.9737	0.9783	0.9755	0.9768
		32	200	0.9794	0.9798	0.1245	0.1199	0.9773	0.9815	0.9790	0.9799
			300	0.9869	0.9875	0.0906	0.0863	0.9859	0.9884	0.9870	0.9875
		64	200	0.9876	0.9878	0.0907	0.0867	0.9862	0.9888	0.9873	0.9878
			300	0.9919	0.9920	0.0664	0.0631	0.9909	0.9927	0.9917	0.9920
	0.01	16	200	0.9837	0.9846	0.0921	0.0873	0.9823	0.9856	0.9837	0.9847
			300	0.9888	0.9886	0.0687	0.0642	0.9867	0.9892	0.9879	0.9886
		32	200	0.9907	0.9910	0.0678	0.0650	0.9897	0.9917	0.9906	0.9910
			300	0.9942	0.9942	0.0494	0.0474	0.9934	0.9946	0.9939	0.9942
		64	200	0.9931	0.9920	0.0534	0.0509	0.9909	0.9926	0.9917	0.9920
			300	0.9957	0.9947	0.0402	0.0388	0.9941	0.9951	0.9946	0.9947
0.005	0.001	16	200	0.8695	0.8832	0.4588	0.4422	0.8837	0.8841	0.8789	0.8843
			300	0.9086	0.9124	0.3163	0.3047	0.9106	0.9139	0.9088	0.9131
		32	200	0.9083	0.9116	0.3003	0.2872	0.9089	0.9132	0.9077	0.9124
			300	0.9439	0.9445	0.2015	0.1946	0.9409	0.9455	0.9418	0.9449
		64	200	0.9641	0.9631	0.1790	0.1732	0.9600	0.9649	0.9617	0.9633
			300	0.9795	0.9756	0.1275	0.1245	0.9730	0.9769	0.9746	0.9757
	0.005	16	200	0.9801	0.9798	0.0962	0.0938	0.9783	0.9800	0.9790	0.9799
			300	0.9868	0.9859	0.0719	0.0711	0.9853	0.9860	0.9856	0.9859
		32	200	0.9902	0.9878	0.0671	0.0656	0.9866	0.9885	0.9874	0.9878
			300	0.9926	0.9902	0.0506	0.0505	0.9895	0.9905	0.9900	0.9902
		64	200	0.9929	0.9928	0.0513	0.0505	0.9919	0.9932	0.9925	0.9928
			300	0.9950	0.9942	0.0386	0.0392	0.9935	0.9944	0.9939	0.9942
	0.01	16	200	0.9868	0.9881	0.0618	0.0593	0.9875	0.9882	0.9878	0.9881
			300	0.9895	0.9891	0.0473	0.0466	0.9886	0.9890	0.9888	0.9891
		32	200	0.9920	0.9910	0.0502	0.0493	0.9905	0.9911	0.9908	0.9910
			300	0.9948	0.9926	0.0385	0.0400	0.9921	0.9927	0.9924	0.9926
		64	200	0.9958	0.9934	0.0340	0.0354	0.9930	0.9933	0.9931	0.9934
			300	0.9970	0.9936	0.0269	0.0309	0.9933	0.9935	0.9934	0.9936
0.01	0.001	16	200	0.8553	0.8627	0.4325	0.4168	0.8601	0.8604	0.8568	0.8655
			300	0.9052	0.9073	0.2978	0.2871	0.9051	0.9098	0.9039	0.9091
		32	200	0.9348	0.9389	0.2336	0.2243	0.9356	0.9388	0.9361	0.9393
			300	0.9610	0.9610	0.1677	0.1620	0.9582	0.9614	0.9594	0.9611
		64	200	0.9513	0.9538	0.1851	0.1803	0.9501	0.9549	0.9517	0.9541
			300	0.9744	0.9766	0.1296	0.1267	0.9745	0.9776	0.9758	0.9767
	0.005	16	200	0.9818	0.9835	0.0866	0.0833	0.9820	0.9840	0.9829	0.9836
			300	0.9851	0.9886	0.0672	0.0643	0.9879	0.9889	0.9884	0.9886
		32	200	0.9898	0.9904	0.0639	0.0621	0.9893	0.9909	0.9901	0.9905
			300	0.9919	0.9896	0.0480	0.0479	0.9890	0.9896	0.9893	0.9896
		64	200	0.9929	0.9904	0.0471	0.0475	0.9897	0.9905	0.9901	0.9904
			300	0.9957	0.9923	0.0363	0.0382	0.9918	0.9922	0.9919	0.9923
	0.01	16	200	0.9885	0.9891	0.0561	0.0558	0.9883	0.9895	0.9888	0.9891
			300	0.9923	0.9907	0.0426	0.0438	0.9899	0.9910	0.9904	0.9907
		32	200	0.9912	0.9896	0.0492	0.0496	0.9892	0.9896	0.9892	0.9896
			300	0.9940	0.9910	0.0377	0.0394	0.9907	0.9904	0.9905	0.9910
		64	200	0.9959	0.9931	0.0309	0.0341	0.9924	0.9935	0.9929	0.9931
			300	0.9970	0.9936	0.0247	0.0304	0.9928	0.9940	0.9934	0.9936

Table-3: Double layer 80:20

alpha (α)	Lr	HL	Epochs	Tr_Acc	Te_Acc	Tr_Loss	Te_Loss	M_Prec	M_rec	M_F1	We_F1
				Performance metrics							
0.001	0.001	16	200	0.9191	0.9323	0.2500	0.2287	0.9304	0.9341	0.9304	0.9321
			300	0.9598	0.9669	0.1470	0.1330	0.9652	0.9676	0.9660	0.9669
		32	200	0.9740	0.9773	0.1408	0.1287	0.9764	0.9760	0.9762	0.9773
			300	0.9852	0.9861	0.0913	0.0843	0.9853	0.9857	0.9855	0.9861
		64	200	0.9865	0.9825	0.0771	0.0723	0.9805	0.9829	0.9816	0.9825
			300	0.9930	0.9900	0.0509	0.0502	0.9887	0.9902	0.9894	0.9901
	0.005	16	200	0.9907	0.9932	0.0451	0.0458	0.9929	0.9934	0.9931	0.9932
			300	0.9938	0.9924	0.0336	0.0402	0.9921	0.9926	0.9924	0.9924
		32	200	0.9961	0.9940	0.0295	0.0342	0.9937	0.9938	0.9938	0.9940
			400	0.9974	0.9940	0.0193	0.0339	0.9937	0.9940	0.9938	0.9940
		64	300	0.9973	0.9924	0.0153	0.0357	0.9921	0.9927	0.9924	0.9924
			400	0.9978	0.9916	0.0120	0.0374	0.9914	0.9919	0.9916	0.9916
	0.01	16	200	0.9936	0.9920	0.0269	0.0338	0.9913	0.9916	0.9914	0.9920
			400	0.9966	0.9940	0.0177	0.0322	0.9937	0.9941	0.9939	0.9940
		32	300	0.9974	0.9940	0.0156	0.0350	0.9936	0.9941	0.9938	0.9940
			400	0.9975	0.9940	0.0130	0.0371	0.9936	0.9938	0.9937	0.9940
		64	300	0.9986	0.9940	0.0106	0.0383	0.9937	0.9938	0.9937	0.9940
			400	0.9984	0.9940	0.0080	0.0419	0.9938	0.9940	0.9939	0.9940
0.005	0.001	16	200	0.9055	0.9104	0.2957	0.2717	0.9065	0.9101	0.9080	0.9107
			300	0.9345	0.9399	0.1980	0.1834	0.9365	0.9400	0.9379	0.9401
		32	200	0.9711	0.9713	0.0860	0.0855	0.9690	0.9702	0.9696	0.9714
			300	0.9810	0.9757	0.0620	0.0703	0.9737	0.9753	0.9744	0.9758
		64	200	0.9841	0.9789	0.0566	0.0638	0.9772	0.9788	0.9779	0.9789
			300	0.9881	0.9813	0.0449	0.0531	0.9798	0.9810	0.9804	0.9813
	0.005	16	200	0.9788	0.9765	0.0646	0.0787	0.9752	0.9761	0.9756	0.9765
			300	0.9853	0.9837	0.0488	0.0666	0.9829	0.9836	0.9833	0.9837
		32	200	0.9891	0.9789	0.0405	0.0676	0.9774	0.9781	0.9778	0.9789
			300	0.9938	0.9833	0.0287	0.0614	0.9823	0.9831	0.9827	0.9833
		64	200	0.9958	0.9920	0.0245	0.0399	0.9913	0.9921	0.9917	0.9920
			300	0.9974	0.9936	0.0189	0.0363	0.9932	0.9938	0.9935	0.9936
	0.01	16	200	0.9838	0.9837	0.0482	0.0648	0.9827	0.9832	0.9829	0.9837
			300	0.9908	0.9877	0.0319	0.0535	0.9870	0.9871	0.9871	0.9877
		32	200	0.9940	0.9853	0.0260	0.0573	0.9841	0.9848	0.9845	0.9853
			300	0.9963	0.9857	0.0187	0.0553	0.9844	0.9857	0.9850	0.9857
		64	200	0.9976	0.9900	0.0165	0.0515	0.9899	0.9900	0.9899	0.9900
			400	0.9989	0.9936	0.0095	0.0507	0.9933	0.9935	0.9934	0.9936
0.01	0.001	16	200	0.8816	0.8913	1.1528	1.0311	0.8863	0.8866	0.8864	0.8912
			300	0.9176	0.9251	0.6464	0.6424	0.9226	0.9208	0.9215	0.9249
		32	200	0.9653	0.9701	0.1752	0.1972	0.9673	0.9705	0.9687	0.9703
			300	0.9771	0.9761	0.1008	0.1443	0.9744	0.9759	0.9751	0.9761
		64	200	0.9848	0.9817	0.0481	0.0887	0.9803	0.9816	0.9810	0.9817
			300	0.9908	0.9841	0.0296	0.0856	0.9832	0.9837	0.9835	0.9841
	0.005	16	200	0.9717	0.9673	0.1121	0.1419	0.9654	0.9665	0.9659	0.9674
			300	0.9788	0.9717	0.0755	0.1127	0.9698	0.9710	0.9704	0.9718
		32	200	0.9887	0.9777	0.0405	0.1490	0.9766	0.9775	0.9770	0.9777
			300	0.9920	0.9805	0.0294	0.1330	0.9796	0.9806	0.9801	0.9805
		64	200	0.9950	0.9865	0.0183	0.1700	0.9861	0.9864	0.9862	0.9865
			400	0.9976	0.9892	0.0101	0.1561	0.9887	0.9895	0.9891	0.9893
	0.01	16	200	0.9785	0.9721	0.0844	0.1258	0.9701	0.9705	0.9703	0.9721
			300	0.9850	0.9749	0.0573	0.1060	0.9737	0.9732	0.9734	0.9749
		32	200	0.9927	0.9849	0.0292	0.0933	0.9848	0.9838	0.9843	0.9849
			300	0.9955	0.9841	0.0183	0.0840	0.9836	0.9834	0.9835	0.9841
		64	200	0.9977	0.9885	0.0121	0.1070	0.9883	0.9875	0.9879	0.9884
			300	0.9981	0.9888	0.0058	0.1088	0.9887	0.9883	0.9885	0.9888

Table-4: Double layer 70:30

alpha (α)	Lr	HL	Epochs	Performance metrics							
				Tr_Acc	Te_Acc	Tr_Loss	Te_Loss	M_Prec	M_rec	M_F1	We_F1
0.001	0.001	16	200	0.9192	0.9225	0.2549	0.2470	0.9188	0.9236	0.9195	0.9228
			300	0.9653	0.9631	0.1364	0.1346	0.9601	0.9629	0.9612	0.9632
		32	200	0.9975	0.9780	0.1090	0.1071	0.9760	0.9788	0.9772	0.9780
			300	0.9868	0.9857	0.0724	0.0730	0.9846	0.9858	0.9851	0.9857
		64	200	0.9890	0.9867	0.0781	0.0770	0.9860	0.9870	0.9864	0.9867
			300	0.9926	0.9896	0.0534	0.0546	0.9891	0.9897	0.9894	0.9896
	0.005	16	200	0.9918	0.9907	0.0440	0.0429	0.9900	0.9902	0.9901	0.9907
			300	0.9947	0.9923	0.0341	0.0372	0.9916	0.9918	0.9917	0.9923
		32	200	0.9958	0.9923	0.0290	0.0339	0.9920	0.9919	0.9919	0.9923
			400	0.9974	0.9928	0.0181	0.0313	0.9925	0.9925	0.9926	0.9928
		64	300	0.9974	0.9939	0.0142	0.0279	0.9937	0.9935	0.9936	0.9939
			400	0.9982	0.9936	0.0107	0.0287	0.9935	0.9936	0.9937	0.9936
	0.01	16	200	0.9944	0.9881	0.0328	0.0414	0.9878	0.9875	0.9876	0.9881
			400	0.9972	0.9902	0.0211	0.0407	0.9899	0.9899	0.9899	0.9902
		32	300	0.9977	0.9939	0.0170	0.0336	0.9939	0.9935	0.9937	0.9939
			400	0.9973	0.9902	0.0147	0.0376	0.9905	0.9891	0.9898	0.9902
		64	300	0.9960	0.9942	0.0118	0.0281	0.9940	0.9941	0.9940	0.9942
			400	0.9989	0.9939	0.0069	0.0306	0.9936	0.9938	0.9937	0.9939
0.005	0.001	16	200	0.8956	0.8959	0.2993	0.3191	0.8913	0.8914	0.8913	0.8960
			300	0.9235	0.9222	0.2055	0.2151	0.9184	0.9187	0.9185	0.9223
		32	200	0.9627	0.9626	0.1100	0.1121	0.9621	0.9604	0.9611	0.9625
			300	0.9766	0.9737	0.0790	0.0867	0.9731	0.9724	0.9727	0.9737
		64	200	0.9840	0.9785	0.0615	0.0661	0.9767	0.9775	0.9771	0.9785
			300	0.9893	0.9843	0.0474	0.0566	0.9834	0.9837	0.9835	0.9843
	0.005	16	200	0.9646	0.9618	0.1049	0.1090	0.9593	0.9615	0.9603	0.9619
			300	0.9771	0.9705	0.0665	0.0743	0.9688	0.9696	0.9692	0.9706
		32	200	0.9894	0.9814	0.0351	0.0547	0.9804	0.9800	0.9802	0.9814
			300	0.9931	0.9835	0.0269	0.0447	0.9830	0.9823	0.9826	0.9835
		64	200	0.9953	0.9867	0.0211	0.0481	0.9861	0.9865	0.9863	0.9867
			300	0.9962	0.9888	0.0162	0.0447	0.9884	0.9888	0.9886	0.9889
	0.01	16	200	0.8989	0.9007	0.3445	0.3578	0.8952	0.8960	0.8955	0.9007
			300	0.9247	0.9257	0.2278	0.2390	0.9218	0.9228	0.9223	0.9257
		32	200	0.9641	0.9689	0.1011	0.0867	0.9671	0.9670	0.9670	0.9689
			300	0.9737	0.9750	0.0767	0.0692	0.9740	0.9738	0.9738	0.9750
		64	200	0.9879	0.9793	0.0489	0.0617	0.9779	0.9782	0.9780	0.9793
			300	0.9918	0.9835	0.0376	0.0538	0.9824	0.9829	0.9826	0.9836
0.01	0.001	16	200	0.8805	0.8834	0.9791	1.0165	0.8777	0.8782	0.8777	0.8836
			300	0.9120	0.9089	0.5839	0.6141	0.9040	0.9051	0.9044	0.9092
		32	200	0.9588	0.9580	0.2104	0.2673	0.9557	0.9569	0.9562	0.9581
			300	0.9674	0.9647	0.1437	0.2119	0.9629	0.9637	0.9633	0.9647
		64	200	0.9832	0.9753	0.0517	0.1286	0.9738	0.9743	0.9741	0.9753
			300	0.9902	0.9785	0.0335	0.1135	0.9771	0.9776	0.9773	0.9785
	0.005	16	200	0.9707	0.9732	0.1144	0.1416	0.9721	0.9722	0.9721	0.9732
			300	0.9785	0.9774	0.0783	0.1209	0.9767	0.9769	0.9767	0.9774
		32	200	0.9898	0.9841	0.0336	0.0989	0.9841	0.9830	0.9835	0.9841
			300	0.9941	0.9851	0.0209	0.0932	0.9850	0.9841	0.9845	0.9851
		64	200	0.9975	0.9862	0.0141	01257	0.9857	0.9856	0.9856	0.9862
			300	0.9980	0.9865	0.0095	0.1227	0.9859	0.9861	0.9860	0.9865
	0.01	16	200	0.9125	0.9156	0.2300	0.2471	0.9123	0.9169	0.9125	0.9172
			300	0.9383	0.9373	0.1757	0.1943	0.9359	0.9383	0.9358	0.9379
		32	200	0.9937	0.9833	0.0290	0.1044	0.9830	0.9822	0.9826	0.9833
			300	0.9959	0.9849	0.0194	0.1014	0.9846	0.9840	0.9843	0.9849
		64	200	0.9981	0.9891	0.0096	0.1274	0.9887	0.9888	0.9888	0.9891
			300	0.9986	0.9907	0.0060	0.1264	0.9905	0.9903	0.9904	0.9907
